# Association Between Area-Level Deprivation and Cardio-Metabolic Risk Factors Among the Adult Population in Russia

**DOI:** 10.3390/ijerph22040594

**Published:** 2025-04-10

**Authors:** Anastasia A. Zelenina, Svetlana A. Shalnova, Oksana M. Drapkina

**Affiliations:** Federal State Institution, National Medical Research Center for Therapy and Preventive Medicine, Ministry of Healthcare of the Russian Federation, Petroverigsky per., 10, Building 3, Moscow 101990, Russia; sshalnova@gnicpm.ru (S.A.S.); odrapkina@gnicpm.ru (O.M.D.)

**Keywords:** deprivation index, cardio-metabolic risk factors, prehypertension, prediabetes, contextual factors

## Abstract

Background: Cardiovascular diseases have been the leading cause of death in the Russian population to date. Methods: Using generalized estimating equations, we examined the links of area-level socio-economic and environmental deprivation with cardiovascular disease risk factors in the adult population as a whole, as well as in men and women separately. Results: People living in more economically deprived areas had 61 percent higher odds of being obese (Q4: odds ratio (OR) 1.61; 95% confidence interval (CI): 1.20–2.16), 2.32 times higher odds of having chronic kidney disease (OR 2.32; 95% CI: 1.56–3.44), up to 57 percent higher odds of having hyperuricemia (OR 1.57; 95% CI: 1.31–1.88), and up to 80 percent higher odds of having diabetes mellitus (OR 1.80; 95% CI: 1.71–1.89), compared to those in the least deprived areas. Individuals living in the most environmentally deprived areas were associated with higher odds of hypertension (OR 1.37; 95% CI: 1.19–1.57) and these associations persisted for both when considering men (OR 1.38; 95% CI: 1.19–1.61) and women (OR 1.37; 95% CI: 1.14–1.65) separately. Conclusions: This is the first study to examine the relationship of area characteristics with cardio-metabolic risk factors such as elevated blood pressure and prediabetes, taking into account individual characteristics among the Russian population.

## 1. Introduction

Cardiovascular diseases (CVD) are the leading cause of death in the Russian population at present. According to the Russian Federal State Statistics Service, the death rate from CVD in 2019 was 573 cases per 100,000 population [1]. In men, the mortality rate from CVD in 2019 was 578 cases per 100,000 population, in women—307 cases per 100,000 population. In 2019, the Russian Federation Ministry of Health initiated the federal project “Fight against cardiovascular diseases” in order to preserve and extend the lives of people fighting cardiovascular diseases [2]. The project aims to reduce mortality from diseases of the circulatory system to 450 cases per 100,000 population by 2024 and provides a set of measures aimed at timely identification of risk factors and reducing the risk of their development.

Arterial hypertension (HTN), elevated blood pressure, diabetes mellitus (DM), prediabetes, hyperuricemia (HUA), chronic renal failure, and obesity are risk factors for CVD [3,4,5,6,7,8].

For the most complete understanding of the mechanisms of development of CVD risk factors, it is necessary to take into account the impacts of indicators that reflect the socio-economic and environmental inequality of the population, which contributes to a more competent approach in the development of programs for the prevention and control of non-communicable diseases as well as public health programs [9,10,11,12].

Indicators of socio-economic and environmental inequality can be divided into regional and individual categories. Most of the studies that examine the Russian population mainly use only individual indicators, such as income, education, and marital status [13,14,15]. The relationships between regional indicators and awareness of HTN [16], as well as associations with mental health [17], individual food preferences [18], and alcohol consumption [19], were also being studied. Unfortunately, the influence of area-level socio-economic and environmental factors on cardio-metabolic parameters such as elevated blood pressure and prediabetes in the Russian population remains insufficiently studied. Therefore, this study aimed to explore the links between the area-level socio-economic and environmental indicators and the CVD risk factors in the adult population of Russia as a whole, as well as in men and women separately. We hypothesize that rising levels of area-level deprivation will indicate an increase in the odds of the occurrence of cardio-metabolic risk factors.

## 2. Materials and Methods

### 2.1. Data Sources

Data for our study were obtained from the cross-sectional phase of the study *Epidemiology of Cardiovascular Risk Factors and Diseases in Regions of the Russian Federation* (ESSE-RF). The ESSE-RF is a study initiated in 2012 which covers 13 regions of Russia. A total of 21,921 participants (men and women) aged 25–64 years, and from every region, were included in our study. The detailed data collection methods have been previously published [20].

The missing values in the dataset were the following: blood glucose (3.47%), hypoglycemic medications (0.62%), systolic blood pressure (SBP) (0.16%), diastolic blood pressure (DBP) (0.16%), height (0.63%), weight (0.23%), creatinine (3.46%), uric acid (3.5%), income (0.69%), education (0.1%), sugar intake (2.15%), salt intake (0.33%), and smoking status (0.15%). The multiple imputation approach was used to impute values for the missing data [21].

### 2.2. Individual-Level Variables

Age, sex, marital status, income, educational level attained, smoking status (three categories: never smoker, former smoker, and current smoker), alcohol drinking status (three categories: never, moderate, and hard-drinking), place of residence (urban and rural), taking antihypertensive and antidiabetic medications, as well as dietary habits (consumption of sugar, salt, milk fat, vegetables, and fruits) were collected via self-report.

Respondents who smoked at least one cigarette per day or who had given up smoking less than a year ago, were considered smokers. For men, drinking less than 168 mL, and for women, drinking less than 84 mL, of pure alcohol per week was considered moderate.

The level of income was assessed by answers to the questions “What part of your income is usually spent on food?”, “Choose the statement that most accurately describes the financial capabilities of your family”, and “How do you assess the well-being of your family compared to others?”. The “poorest” answer was assigned 1 point; the “richest” answer was assigned 5 points. The scores for the three questions were summed. The “low income” category included respondents with a score of 3 to 7; in the category “middle income”—those with a sum of 8 to 10; in the category of “high income”—those with an amount of 11 to 15.

A daily consumption of sugar as a raw product (i.e., granulated sugar and refined sugar), or in the form of jam, at or above the amount of 50 g (≥12 teaspoons) was considered excessive. Lack of daily consumption of fresh vegetables and fruits was considered an insufficiency. The presence of more than two high-fat dairy products in the diet was assessed as excess consumption of milk fat. Excessive salt intake was defined as adding salt to prepared foods and/or daily intake of salty foods.

The blood pressure measurement was carried out in a sitting position, using the right hand, and employing an Omron device, after a rest of 5 min. The blood pressure level was measured twice, with an interval of approximately 2–3 min.

### 2.3. Cardio-Metabolic Risk Factors

In our study we looked at the following cardio-metabolic risk factors: HUA, HTN, elevated blood pressure, DM, prediabetes, obesity, and chronic kidney disease (CKD). HUA was defined as serum uric acid (SUA) SUA > 420 μmol/L for men and >360 μmol/L for women. HTN was defined as an SBP ≥ 140 mmHg and/or DBP ≥ 90 mmHg, and/or the current use of antihypertensive medication. Elevated blood pressure was defined as an SBP from 120–139 mmHg, or DBP from 80–89 mmHg, without taking antihypertensive medications. DM was defined as a fasting glucose level ≥ 7 mmol/L, and/or a self-reported physician diagnosis, and/or the current use of glucose-lowering medications. Prediabetes was defined as a fasting glucose level between 5.6 mmol/L and 6.9 mmol/L without having been previously diagnosed with diabetes/high blood sugar levels or taking any diabetes medication. Obesity was defined as a body mass index (BMI) ≥ 30 kg/m^2^. Glomerular filtration rate (GFR) was estimated using the Chronic Kidney Disease Epidemiology Collaboration equation [22]. Individuals with a GFR < 60 mL/min/1.73 m^2^ were considered to have CKD.

### 2.4. Area-Level Deprivation Index

The Russian deprivation index (RDI) was used to measure the level of deprivation. Indicators for the index were obtained from official statistical publications (Table S2) [23]. Principal component analysis was used to create the index. The RDI includes 17 socio-economic and environmental indicators (Tables S1 and S2). The values of the deprivation index and its components were divided into four quartiles. The first quartile (Q1) refers to the least deprived areas; the fourth quartile (Q4) refers to the most deprived areas. The index measures general deprivation, and its components measure social, economic, and environmental deprivation, respectively (Figures S1–S4). Further details on the creation of the RDI have been described in a previous publication [24].

### 2.5. Statistical Analysis

Continuous variables are presented as median (Med) and interquartile range (IQR). Binary and categorical variables are presented as frequencies and percentages. Kruskal–Wallis and Pearson’s χ^2^ tests were used to test for differences between quintiles of deprivation in continuous and categorical variables, respectively.

To assess the link of deprivation with the mentioned CVD risk factors, as well as baseline blood pressure (SBP and DBP), creatinine, fasting glucose, uric acid levels, and BMI, generalized estimating equations with an independent correlation structure and that incorporated the Huber–White standard error were used [25]. For binary dependent variables, a binary logistic model of generalized estimation equations was used; for continuous dependent variables, a linear model was used. Odds ratios (ORs) and regression coefficients (β), as well as 95% confidence intervals (CIs), were calculated for logistic and linear models respectively, for the overall population and for subgroups stratified by sex.

We conducted a series of three binary logistic models of generalized estimation equations for each of the four types of deprivation (general, social, economic, and environmental) (Tables S14–S16). Model 0 estimated the association between deprivation (general, social, economic, and environmental) and the CVD risk factors of interest (HTN, elevated blood pressure, DM, prediabetes, HUA, and CKD). Model 1 added variables that the random forest algorithm identified as “important”. The random forest algorithm was used to select important regional (the index and its components) and individual predictors (age, sex, place of residence, marital status, income and education level, smoking and alcohol drinking status, and dietary habits (consumption of sugar, salt, dairy fats, vegetables, and fruit)) to reduce the model and evaluate its predictive performance (Tables S3–S5). Finally, in Model 2, individual predictors (age, sex (for total population), marital status, place of residence, income and education level, smoking and alcohol drinking status, and dietary habits (consumption of sugar, salt, dairy fats, vegetables, and fruit) were added to Model 0.

All linear models of generalized estimation equations were adjusted for sex, age, marital status, income and education levels, smoking and alcohol drinking status, place of residence, and dietary habits (consumption of sugar, salt, dairy fats, vegetables, and fruits) (Table S17). Additionally, models for estimating the differences in the mean SBP and DBP levels were adjusted with respect to the population taking antihypertensive medications; models for estimating the differences in the mean of fasting glucose level were adjusted for those taking antidiabetic medications.

For simplicity of understanding, the results of exploring the relationships of social, economic, and environmental deprivation with CVD risk factors are described in context of the statistically significant relationships in Q4 (the most deprived areas), or for associations that had a statistically significant linear dependence.

All statistical analyses were conducted using R 4.3.1. A *p* value less than 0.05 was taken to indicate statistical significance. The following packages were used: “Amelia”, “gee”, “Boruta”, and “stats”. All models were checked for multicollinearity using the function vif() from the package “car”.

## 3. Results

### 3.1. Baseline Characteristics

Table S6 shows that 9965 respondents lived in the most generally deprived areas (Q4), of whom 3564 (35.77%) were men and 6401 (64.23%) were women; the median [IQR] age of the respondents at baseline was 49 [37;57] years. The most socially deprived areas had 5395 respondents, of whom 1848 (34.25%) were men and 3547 (65.75%) were women (Table S7). The median [IQR] age of the respondents at baseline was 50 [39;57] years. The respondents with lower levels of education and lower incomes lived in areas which were more generally and socially deprived.

The most economically deprived areas were associated with 1585 respondents, of whom 580 (36.59%) were men and 1005 (63.41%) were women (Table S8). The median age of the respondents at baseline was 53 [42;59] years. The most environmentally deprived areas (Q4) were associated with 10,072 respondents; of them, 4053 (40.24%) were men and 6019 (59.76%) were women (Table S9). The median [IQR] age of the respondents at baseline was 48 [36;56] years. There were health differences between the areas; people living in the more economically and environmentally deprived areas were more likely to report having cardiovascular risk factors (high blood pressure, creatinine, glucose, and uric acid levels) at baseline compared with people in the areas described as the least deprived.

### 3.2. Association of General Deprivation with HTN, Elevated Blood Pressure, Obesity, DM, Prediabetes, HUA, and CKD

The relationships of general deprivation with obesity, prediabetes, and CKD were not significant in analyses separately considering men and women, as well as in the total population (Table 1).

### 3.3. Association of Social Deprivation with HTN, Elevated Blood Pressure, Obesity, DM, Prediabetes, HUA, and CKD

In Model 1, living in the most socially deprived areas was associated with lower odds of having HUA (Q4: OR 0.74; 95% CI: 0.58–0.95), and this association persisted in both men and women separately (Table 2). In Model 1, both the total population and the men living in the most socially deprived areas (Q4) had significantly lower odds of having prediabetes, compared to those in the least deprived areas (Q1).

### 3.4. Association of Economic Deprivation with HTN, Elevated Blood Pressure, Obesity, DM, Prediabetes, HUA, and CKD

In Model 1, people living in more economically deprived areas had 61 percent higher odds of being obese (Q4: OR 1.61; 95% CI: 1.20–2.16), 2.32 times higher odds of having CKD (Q4: OR 2.32; 95% CI: 1.56–3.44), up to 57 percent higher odds of having HUA (Q4: OR 1.57; 95% CI: 1.31–1.88), and up to 80 percent higher odds of having diabetes (Q4: OR 1.80; 95% CI: 1.71–1.89), compared to those in the least deprived areas (Q1) (Table 3).

There is an increasing trend in the odds of having HTN and prediabetes, depending on the level of economic deprivation of the area (from less deprived (Q2) to the most deprived (Q4)), as compared to the least deprived areas (Q1), in both the total population and among women (Table 3). Among men, there is only an increasing trend in the odds of having HTN, depending on the level of economic deprivation of the area (from less deprived (Q2) to the most deprived (Q4)), as compared to the least deprived areas (Q1).

In Model 1, women living in more economically deprived areas had 71 percent higher odds of being obese (Q4: OR 1.71; 95% CI: 1.28–2.30), 2.83 times higher odds of having CKD (Q4: OR 2.83; 95% CI: 1.41–5.68), up to 77 percent higher odds of having HUA (Q4: OR 1.77; 95% CI: 1.40–2.25), and up to 95 percent higher odds of having diabetes (Q4: OR 1.95; 95% CI: 1.69–2.25), compared to those in the least deprived areas (Q1) (Table 3).

In Model 2, men living in more economically deprived areas had 1.42 times higher odds of having obesity (Q4: OR 1.42; 95% CI: 1.05–1.92). (Model 1 was not constructed, due to the fact that the random forest algorithm identified the economic component of the index as “unimportant” for obesity in men.)

### 3.5. Association of Environmental Deprivation with HTN, Elevated Blood Pressure, Obesity, DM, Prediabetes, HUA, and CKD

In Model 1, the total population living in the most environmentally deprived areas (Q4) was associated with higher odds of HTN, and this link persisted in both men and women separately (Table 4). Both the total population and men living in the most environmentally deprived areas (Q4) had higher odds of having prediabetes. Among women, there is an increasing trend in the odds of having prediabetes, depending on the level of environmental deprivation of the area (from less deprived (Q2) to the most deprived (Q4)), as compared to the least deprived areas (Q1). Both the total population and women living in the most environmentally deprived areas (Q4) had higher odds of having elevated blood pressure and HUA, compared to those in the least deprived areas (Q1).

### 3.6. Association of General Deprivation with Blood Pressure, Creatinine, Fasting Glucose, Uric Acid Levels, and BMI

The relationships of general deprivation with blood pressure, creatinine, fasting glucose, uric acid levels, and BMI were not significant, as to both women and men, as well as in the total population (Table S10).

### 3.7. Association of Social Deprivation with Blood Pressure, Creatinine, Fasting Glucose, Uric Acid Levels, and BMI

Table S11 shows the association of social deprivation with fasting glucose and uric acid levels. Individuals living in the least versus most socially deprived areas were found to have lower mean fasting glucose (Q4 versus Q1 = −0.54 mmol/L, 95% CI: −0.90; −0.17) and lower mean uric acid levels (Q4 versus Q1 = −19.26 μmol/L, 95% CI: −29.67; −8.86). Also, both men and women living in the least (versus most) socially deprived areas were found to have lower mean fasting glucose and uric acid levels.

### 3.8. Association of Economic Deprivation with Blood Pressure, Creatinine, Fasting Glucose, Uric Acid Levels, and BMI

Individuals living in the least (versus most) economically deprived areas were found to have higher mean SBP (Q4 versus Q1 = 11.02 mmHg, 95% CI: 8.87; 13.17), DBP (Q4 versus Q1 = 4.07 mmHg, 95% CI: 2.70; 5.45), creatinine (Q4 versus Q1 = 0.05 mg/dL, 95% CI: 0.01; 0.09), fasting glucose (Q4 versus Q1 = 1.0 mmol/L, 95% CI: 0.81; 1.18), uric acid levels (Q4 versus Q1 = 26.81 μmol/L, 95% CI: 17.91; 35.71), and BMI (Q4 versus Q1 = 1.26 kg/m^2^, 95% CI: 0.50; 2.01), and these associations persisted in both men and women separately (Table S12).

### 3.9. Association of Environmental Deprivation with Blood Pressure, Creatinine, Fasting Glucose, Uric Acid Levels, and BMI

Individuals living in the least (versus most) environmentally deprived areas were found to have higher mean SBP (Q4 versus Q1 = 5.70 mmHg, 95% CI: 3.06; 8.34), DBP (Q4 versus Q1 = 2.73 mmHg, 95% CI: 1.54; 3.92), fasting glucose (Q4 versus Q1 = 0.34 mmol/L, 95% CI: 0.05; 0.62), and uric acid levels (Q4 versus Q1 = 20.22 μmol/L, 95% CI: 3.94; 36.51), and these associations persisted among women (Table S13). Men living in the least (versus most) environmentally deprived areas were only found to have higher mean SBP and DBP.

## 4. Discussion

Our findings show that a rising level of economic deprivation was linked with elevated average fasting glucose, SBP, and DBP levels in both genders. Furthermore, there were statistically significant associations of HTN and prediabetes with economic deprivation among both women and men, as well as in the total population. In women, the rising level of environmental deprivation was linked to increased odds of having prediabetes. In addition, in women, there was a statistically significant association between the average uric acid level and environmental deprivation.

Our study found statistically significant positive associations of economic deprivation with HTN, obesity, DM, and CKD among both women and men, as well as in the total population. The economic component of our index includes indicators of income (percentage of people below a low-income threshold in the total population) and housing conditions (stove heating, the absence of hot water supply, sewerage system, and central sewerage system). Therefore, it can be said that these results are analogous to the findings of Lucumi et al. [26], who confirmed that high income inequality was linked, in women, with higher SBP and greater odds of HTN. Diez-Roux et al. [27] found positive relationships in women between area-level income inequality and HTN and obesity. Our findings are supported by a study conducted in Canada which found that participants living in low-income areas had a 1.5 times higher prevalence of diabetes (rate ratio (RR) 1.52; 95% CI: 1.29–1.80) [28]. This study also showed that both women and men living in low-income areas had significantly higher levels of obesity. Tang et al. [29] found that in the medium gross domestic product (GDP)-per capita areas, the prevalence of DM was the highest in both genders. GDP per capita indicates the level of economic development among regions. Conversely, another study [30] found that the prevalence of HTN was the highest in the high GDP-per capita areas in both men and women. A few studies [31,32] found that neighborhood poverty was positively associated with end-stage renal disease incidence.

Housing conditions are a crucial aspect that affects health [33]. It is important, however, to point out that housing conditions are directly related to the economic status of the population. Poor housing conditions have both direct and indirect impacts on human health. The outcomes of direct influence can include infectious diseases [34], trauma, and poisoning [35]. Among the examples of indirect influence, several studies found an association between poor housing conditions and poor mental health [36,37]; it is well known that poor mental health is a trigger for the development of cardiovascular diseases [38,39,40,41,42], which emphasizes once again the importance of considering housing conditions when studying the mechanisms of cardiovascular disease development.

In the current study, there were statistically significant positive associations of environmental deprivation with HTN, elevated blood pressure, prediabetes, and HUA in the total population. The environmental component of our index includes such indicators as transport-related emissions and emissions from stationary sources (NO_2_, SO_2_, CO). Thus, these results are similar to the findings of Yang et al. [43], who conducted a systematic review and meta-analysis and found a significant association between long-term exposure to NO_2_ and DBP. Mei et al. [44] found that, in both genders, long-term exposure to SO_2_ was linked with elevated DBP. Zhang et al. [45] indicated that long-term exposure to SO_2_, NO_2_, or CO was associated with elevated SBP and DBP. Liu et al. [46] suggested that exposure to SO_2_, NO_2_, or CO was also linked with HTN morbidity. Howell et al. [47] reported that increasing NO_2_ exposure was positively associated with HTN (OR 1.02; 95% CI: 1.01–1.03). Yang et al. [48] found that the associations of SO_2_ and NO_2_ with elevated blood pressure were significant, and increasing SO_2_ exposure was significantly associated with SBP among overweight adults. Yang et al. [49] found that exposure to SO_2_ (OR 1.11; 95% CI: 1.00–1.25) or NO_2_ (OR 1.18; 95% CI: 1.05–1.32) was significantly associated with an increased prevalence of elevated blood pressure. Feizi et al. [50] found that exposure to NO_2_ was significantly associated with an increased risk of the development of prediabetes in participants with normal glucose tolerance (hazard ratio (HR) 1.118; 95% CI: 1.085–1.152). A few studies have explored the effects of air pollution on glucose concentrations, and positive associations are generally reported. For instance, Chen et al. [51] found that increased exposure to NO_2_ or SO_2_ was significantly linked with elevated fasting blood glucose levels. This study also revealed that the effects of NO_2_ or SO_2_ on fasting blood glucose were stronger in females than in males. Another study [52] conducted in Southern Israel also showed that NO_2_ and SO_2_ exposure were linked with significantly increased levels of serum glucose. On the other hand, Lucht et al. [53] showed that no association had been found between NO_2_ exposure and blood glucose. A study conducted in Guangzhou, China [54] indicated that increasing NO_2_ (HR 1.43; 95% CI: 1.26–1.61) and SO_2_ (HR 1.23; 95% CI: 1.00–1.51) exposure were positively associated with HUA. Duan et al. [55] also revealed that exposure to NO_2_ was linked with HUA (HR 1.178; 95% CI: 1.125–1.233).

In our study, a link between the most environmentally deprived regions and HTN was established among both men and women. A study conducted in China [56] that included 24,845 participants (12,661 were men) aged 18 to 74 years, however, found significant associations between long-term exposure to SO_2_ and HTN among men (OR 1.19; 95% CI: 1.05–1.34), but not among women (OR 1.03; 95% CI: 0.91–1.89). In addition, there was no association of NO_2_ with the prevalence of HTN among either men or women. Among men and among women, our results did not reveal an association between the most environmentally deprived regions and DM. These results are similar to the findings of Lin et al. [57], who found no relationship between NO_2_ or CO and diabetes, both among women and among men. Eze et al. [58], however, conducted a systematic review and found that the association between NO_2_ exposure and diabetes was stronger in women than in men. In turn, Sohn et al. [59] showed that exposure to SO_2_ was significantly related to the prevalence of type 2 diabetes mellitus among women but not among men.

A number of studies have suggested that one pathophysiology explaining a possible association between air pollutants and cardio-metabolic disorders is linked to an inflammatory response [60,61,62]. Furthermore, this inflammatory response is involved in insulin signaling pathways, and this may disrupt the lipid and glucose metabolism process [63,64,65,66]. In addition, the systemic inflammation may also underpin the association of air pollution with HUA [67].

The environmental component of our index also includes such variables as the number of firefighters involved. Several studies indicate that a positive association exists between exposure to wildfire smoke and cardiovascular disorders [68,69,70,71].

The differences in the accessibility of health services, dietary habits, and environmental factors among different economically and environmentally deprived areas might explain the higher odds of having risk factors for cardiovascular and metabolic diseases found among people living in the more economically and environmentally deprived areas. Since, in the most economically deprived regions, low-income households predominate, they have poorer access to outpatient care [72]. Furthermore, a low-income population has lower adherence to a general medical check-up regime [73,74], which hinders the identification of risk factors for diseases and leads to the detection of diseases at later stages [75,76,77]. Low-income families have been found to be unable to buy healthy food with low salt and sugar due to its high cost [78,79,80]. In addition, low-income families cannot afford to move and live in more environmentally friendly areas [81]. This fact links the economic and environmental well-being of these areas, and is confirmed by our results.

At the moment, we cannot explain why our findings show that living in the most socially deprived areas was linked with lower odds of having HUA (total population, men, and women) or prediabetes (total population and men), as well as decreased average glucose and uric acid levels (total population, men, and women). This may be due to the fact that the social component of the index includes indicators of family structure, such as families with children under the age of 5 years old and families with three or more children (aged 0–18), which suggests that, for the most part, young and multi-child families are living in the most socially deprived regions; accordingly, the health indicators of the young parents may generally be within normal limits. On the other hand, our results can be related to the social heterogeneity of the regions, which may lead to the ecological fallacy [82,83]. However, in any case, owing to a lack of studies in this field among the Russian population, it is necessary to conduct further studies to analyze the influence of area-level social indicators on cardio-metabolic risk factors in more depth. When examining the impact of family structure on the health of parents, it is necessary to take into account the fact that this relationship involves complex biological and psychophysiological mechanisms that are closely related to the context of the country where the study is conducted [84].

This study builds on emerging evidence that economic and environmental deprivation are positively associated with cardio-metabolic risk factors, utilizing the large Russian cohort and cardio-metabolic risk factors adjusted for multiple potential confounders.

Overall, our results are contradictory. On the one hand, it was found that people living in the most economically and environmentally deprived regions were more likely to have some cardio-metabolic risk factors than those living in the least deprived regions; on the other hand, it was found that people living in the most socially deprived regions were less likely to have some risk factors, which suggests that people living in regions which are the most generally deprived will be more likely to have risk factors, given the fact that general deprivation sums up the influences of social, economic, and environmental deprivation. However, our results show that there were no significant associations found between conditions of the highest levels of general deprivation and the cardio-metabolic risk factors, in the adjusted models. Due to the limited amount of research in this field, we cannot know exactly, but assume that these contradictions are due to the fact that the social component of RDI is the first component [24] (the first component explains 24.8% of the total variance, the second component (economic) explains 24.6% of the total variance, and the third component (environmental) explains 24% of the total variance), and its influence may dominate when calculating the general deprivation.

### 4.1. Limitations of This Study

We acknowledge several limitations. First, we cannot take into account how long the participants of the study have been residents of a certain area, due to the absence of this indicator in the database, which may lead to overestimation in the results. Secondly, the association between area-level deprivation and cardio-metabolic risk factors may be confounded by differences in the quality and availability of services in health-care facilities within an area, but these assumptions are not considered in our study due to lack of data. Thirdly, we used data from a cross-sectional study, so we cannot assess the risks of the development of cardio-metabolic outcomes over a period of time.

### 4.2. Implications for Practice and Policy

Our findings point to potential directions for political decisions, such as implementing measures to reduce economic and environmental deprivation at the area level, and provide indicators for public health professionals aiming to develop preventive strategies to address risk factors for cardiovascular and metabolic diseases.

## 5. Conclusions

To the best of our knowledge, this is the first study to examine the relationship of area-level characteristics with cardio-metabolic risk factors such as elevated blood pressure and prediabetes, taking into account individual characteristics, among the Russian population. In the future, the impacts of spatial characteristics on other health outcomes should be studied, and the results used to develop preventive programs and improve the health system in Russia. In general, the results of the current study and future research will enable a scientific approach to management decision-making in public health.

## Figures and Tables

**Table 1:** Association of general deprivation ** with CVD risk factors.

Dependent Variables	Level of Deprivation ***	Total Population			Men			Women		
		Model 0	Model 1	Model 2	Model 0	Model 1	Model 2	Model 0	Model 1	Model 2
		OR (95% CI)	OR (95% CI)	OR (95% CI)	OR (95% CI)	OR (95% CI)	OR (95% CI)	OR (95% CI)	OR (95% CI)	OR (95% CI)
Hypertension	Q1	REF			REF			REF		
	Q2	1.05 (0.36–3.03)	0.97 (0.36–2.60)	0.97 (0.36–2.60)	0.90 (0.32–2.49)	0.84 (0.32–2.23)	0.87 (0.32–2.38)	1.16 (0.39–3.49)	1.07 (0.41–2.81)	1.07 (0.41–2.81)
	Q3	1.04 (0.80–1.34)	1.09 (0.80–1.47)	1.09 (0.80–1.48)	1.22 (1.18–1.27) *	1.18 (1.05–1.34) *	1.21 (1.07–1.37) *	0.92 (0.61–1.38)	1.01 (0.68–1.52)	1.02 (0.68–1.53)
	Q4	1.12 (0.84–1.51)	1.08 (0.78–1.50)	1.08 (0.77–1.50)	1.18 (0.99–1.42)	1.10 (0.87–1.38)	1.15 (0.93–1.43)	1.09 (0.70–1.70)	1.05 (0.69–1.60)	1.05 (0.69–1.62)
Elevated blood pressure	Q1	REF			REF			REF		
	Q2	0.41 (0.21–0.80) *	0.41 (0.22–0.74) *	0.41 (0.22–0.75) *	0.36 (0.17–0.77) *	0.37 (0.19–0.74) *	0.38 (0.19–0.73) *	0.43 (0.23–0.81) *	0.45 (0.25–0.81) *	0.46 (0.25–0.83) *
	Q3	1.01 (0.55–1.85)	0.98 (0.56–1.71)	0.98 (0.56–1.71)	0.85 (0.48–1.51)	0.87 (0.51–1.48)	0.87 (0.53–1.45)	1.12 (0.59–2.14)	1.10 (0.59–2.05)	1.11 (0.60–2.04)
	Q4	0.85 (0.46–1.55)	0.87 (0.49–1.54)	0.88 (0.50–1.56)	0.77 (0.43–1.37)	0.81 (0.48–1.39)	0.81 (0.48–1.36)	0.93 (0.49–1.75)	0.95 (0.51–1.76)	0.96 (0.52–1.79)
Obesity	Q1	REF			REF			REF		
	Q2	1.11 (0.53–2.29)	1.08 (0.61–1.90)	1.07 (0.61–1.91)	0.91 (0.54–1.54)	0.96 (0.60–1.54)	0.96 (0.60–1.54)	1.26 (0.56–2.84)	1.20 (0.66–2.18)	1.18 (0.65–2.15)
	Q3	0.95 (0.73–1.23)	0.99 (0.78–1.25)	0.99 (0.78–1.26)	0.96 (0.79–1.18)	0.96 (0.76–1.22)	0.97 (0.76–1.23)	0.97 (0.71–1.31)	1.00 (0.79–1.26)	1.00 (0.78–1.29)
	Q4	1.18 (0.88–1.57)	1.12 (0.86–1.46)	1.12 (0.86–1.47)	1.01 (0.83–1.23)	1.04 (0.81–1.33)	1.04 (0.81–1.35)	1.26 (0.90–1.78)	1.21 (0.94–1.56)	1.20 (0.92–1.57)
Diabetes mellitus	Q1	REF			REF			REF		
	Q2	1.60 (0.88–2.89)	1.44 (1.01–2.04) *	1.44 (1.02–2.04) *	1.49 (0.82–2.72)	1.40 (0.90–2.19)	1.41 (0.92–2.16)	1.68 (0.95–2.99)	1.49 (1.09–2.03) *	1.47 (1.09–1.99) *
	Q3	1.14 (0.85–1.53)	1.15 (0.90–1.49)	1.12 (0.85–1.48)	1.21 (0.90–1.61)	1.13 (0.91–1.40)	1.09 (0.87–1.37)	1.12 (0.78–1.60)	1.23 (0.88–1.71)	1.21 (0.83–1.77)
	Q4	1.15 (0.96–1.38)	1.02 (0.83–1.25)	1.02 (0.84–1.25)	1.35 (1.00–1.82) *	1.16 (0.94–1.44)	1.14 (0.94–1.38)	1.05 (0.85–1.30)	0.99 (0.76–1.30)	1.00 (0.77–1.28)
Prediabetes	Q1	REF			REF			REF		
	Q2	1.38 (0.39–4.94)	1.35 (0.40–4.60)	1.37 (0.40–4.69)	1.18 (0.32–4.27)	1.16 (0.34–3.91)	1.21 (0.36–4.13)	1.53 (0.42–5.55)	1.57 (0.48–5.13)	1.57 (0.48–5.10)
	Q3	1.17 (0.85–1.60)	1.18 (0.85–1.64)	1.19 (0.86–1.66)	1.14 (0.82–1.59)	1.15 (0.80–1.66)	1.19 (0.82–1.73)	1.12 (0.77–1.62)	1.19 (0.83–1.69)	1.19 (0.83–1.72)
	Q4	0.94 (0.57–1.55)	0.94 (0.57–1.54)	0.96 (0.58–1.57)	1.01 (0.62–1.64)	0.99 (0.59–1.66)	1.02 (0.60–1.73)	0.89 (0.49–1.60)	0.93 (0.55–1.57)	0.94 (0.55–1.59)
Chronic kidney disease	Q1	REF			REF			REF		
	Q2	1.35 (0.47–3.91)	1.22 (0.48–3.08)	1.22 (0.48–3.08)	1.09 (0.51–2.33)	1.12 (0.52–2.44)	1.06 (0.49–2.31)	1.56 (0.44–5.53)	1.47 (0.43–5.07)	1.34 (0.46–3.91)
	Q3	1.08 (0.45–2.61)	1.06 (0.47–2.38)	1.06 (0.47–2.36)	0.92 (0.37–2.29)	0.91 (0.37–2.27)	0.89 (0.37–2.15)	1.21 (0.49–3.00)	1.17 (0.48–2.86)	1.15 (0.51–2.60)
	Q4	1.54 (0.66–3.60)	1.38 (0.63–2.99)	1.37 (0.64–2.96)	1.06 (0.53–2.11)	1.02 (0.49–2.11)	0.95 (0.44–2.07)	1.90 (0.72–5.04)	1.78 (0.69–4.60)	1.63 (0.71–3.77)
Hyperuricemia	Q1	REF			REF			REF		
	Q2	0.76 (0.46–1.24)	0.79 (0.49–1.28)	0.79 (0.49–1.27)	0.51 (0.32–0.82) *	0.58 (0.37–0.93) *	0.59 (0.36–0.94) *	1.00 (0.60–1.68)	0.96 (0.63–1.46)	0.97 (0.64–1.46)
	Q3	1.17 (0.84–1.64)	1.21 (0.82–1.79)	1.21 (0.83–1.78)	1.08 (0.66–1.77)	1.14 (0.69–1.89)	1.14 (0.69–1.90)	1.16 (0.90–1.50)	1.24 (0.95–1.61)	1.23 (0.94–1.62)
	Q4	0.87 (0.59–1.28)	0.92 (0.60–1.43)	0.92 (0.60–1.43)	0.73 (0.54–1.00) *	0.84 (0.60–1.19)	0.83 (0.58–1.20)	1.00 (0.63–1.58)	1.01 (0.61–1.68)	1.02 (0.61–1.70)

Q: quartile; CI: confidence interval; OR: odds ratio; CVD: cardiovascular diseases; REF: reference category. * Boldface indicates statistical significance (*p* < 0.05). ** The Russian deprivation index measures general deprivation, and its components measure social, economic, and environmental deprivation, respectively. *** Q1—the least deprived areas; Q4—the most deprived areas. Model 0 was unadjusted; Model 1 was adjusted for independent variables that the random forest algorithm identified as “important”; Model 2—Model 0 + age, sex (for total population), marital status, income and education level, smoking and alcohol drinking status, place of residence, and dietary habits (consumption of sugar, salt, dairy fats, vegetables, and fruit).

**Table 2:** Association of social deprivation ** with CVD risk factors.

Dependent Variables	Level of Deprivation ***	Total Population			Men			Women		
		Model 0	Model 1	Model 2	Model 0	Model 1	Model 2	Model 0	Model 1	Model 2
		OR (95% CI)	OR (95% CI)	OR (95% CI)	OR (95% CI)	OR (95% CI)	OR (95% CI)	OR (95% CI)	OR (95% CI)	OR (95% CI)
Hypertension	Q1	REF			REF			REF		
	Q2	0.60 (0.30–1.22)	0.63 (0.32–1.23)	0.63 (0.32–1.23)	0.64 (0.29–1.40)	0.63 (0.30–1.33)	0.63 (0.29–1.37)	0.58 (0.30–1.14)	0.64 (0.35–1.18)	0.64 (0.35–1.18)
	Q3	0.78 (0.49–1.22)	0.84 (0.56–1.25)	0.84 (0.56–1.25)	0.93 (0.62–1.39)	0.93 (0.66–1.32)	0.94 (0.65–1.36)	0.69 (0.41–1.15)	0.79 (0.51–1.23)	0.80 (0.51–1.24)
	Q4	0.97 (0.59–1.60)	0.92 (0.59–1.41)	0.92 (0.59–1.41)	1.00 (0.65–1.52)	0.91 (0.59–1.40)	0.93 (0.61–1.43)	0.96 (0.53–1.74)	0.90 (0.56–1.46)	0.91 (0.56–1.46)
Elevated blood pressure	Q1	REF			REF			REF		
	Q2	0.91 (0.42–1.97)	0.85 (0.41–1.79)	0.86 (0.41–1.80)	0.87 (0.38–1.98)	0.86 (0.39–1.90)	0.89 (0.42–1.89)	0.88 (0.41–1.90)	0.82 (0.41–1.66)	0.84 (0.42–1.66)
	Q3	1.22 (0.59–2.53)	1.17 (0.59–2.31)	1.18 (0.60–2.33)	1.10 (0.50–2.43)	1.09 (0.52–2.29)	1.10 (0.53–2.25)	1.30 (0.65–2.60)	1.25 (0.65–2.41)	1.26 (0.66–2.41)
	Q4	1.14 (0.55–2.36)	1.19 (0.60–2.35)	1.20 (0.61–2.37)	1.12 (0.50–2.52)	1.19 (0.57–2.49)	1.20 (0.58–2.47)	1.18 (0.60–2.33)	1.20 (0.63–2.28)	1.22 (0.64–2.29)
Obesity	Q1	REF			REF			REF		
	Q2	0.69 (0.44–1.07)	0.74 (0.53–1.04)	0.74 (0.53–1.04)	0.79 (0.53–1.17)	0.81 (0.58–1.13)	0.81 (0.58–1.14)	0.65 (0.40–1.06)	0.70 (0.50–0.99) *	0.70 (0.50–0.98) *
	Q3	0.79 (0.56–1.11)	0.85 (0.64–1.14)	0.85 (0.64–1.14)	0.81 (0.66–0.99) *	0.84 (0.66–1.06)	0.84 (0.66–1.07)	0.80 (0.52–1.22)	0.88 (0.63–1.24)	0.89 (0.63–1.25)
	Q4	1.13 (0.80–1.60)	1.08 (0.82–1.41)	1.08 (0.82–1.41)	1.04 (0.86–1.27)	1.03 (0.80–1.32)	1.04 (0.81–1.33)	1.17 (0.76–1.80)	1.11 (0.81–1.52)	1.11 (0.81–1.51)
Diabetes mellitus	Q1	REF			REF			REF		
	Q2	0.81 (0.49–1.35)	0.90 (0.64–1.27)	0.88 (0.63–1.23)	0.91 (0.52–1.60)	0.92 (0.61–1.39)	0.91 (0.61–1.36)	0.76 (0.46–1.25)	0.89 (0.64–1.25)	0.89 (0.65–1.21)
	Q3	0.82 (0.47–1.42)	0.90 (0.60–1.35)	0.90 (0.60–1.35)	0.84 (0.50–1.40)	0.84 (0.55–1.27)	0.81 (0.55–1.20)	0.82 (0.45–1.47)	0.98 (0.64–1.50)	0.99 (0.65–1.50)
	Q4	0.80 (0.48–1.33)	0.72 (0.52–1.00) *	0.72 (0.52–0.99) *	1.06 (0.56–1.98)	0.89 (0.60–1.33)	0.87 (0.58–1.31)	0.69 (0.42–1.12)	0.66 (0.47–0.93) *	0.66 (0.48–0.91) *
Prediabetes	Q1	REF			REF			REF		
	Q2	0.47 (0.19–1.15)	0.48 (0.20–1.12)	0.48 (0.20–1.13)	0.43 (0.19–0.96) *	0.44 (0.21–0.94) *	0.46 (0.21–0.97) *	0.49 (0.18–1.30)	0.51 (0.21–1.26)	0.52 (0.21–1.25)
	Q3	0.86 (0.45–1.64)	0.89 (0.49–1.62)	0.90 (0.49–1.64)	0.98 (0.54–1.79)	1.01 (0.57–1.79)	1.03 (0.58–1.82)	0.74 (0.37–1.47)	0.81 (0.42–1.54)	0.81 (0.42–1.55)
	Q4	0.44 (0.21–0.93) *	0.44 (0.22–0.86) *	0.44 (0.22–0.87) *	0.44 (0.25–0.78) *	0.43 (0.25–0.74) *	0.44 (0.25–0.76) *	0.44 (0.16–1.24)	0.45 (0.18–1.09)	0.45 (0.18–1.10)
Chronic kidney disease	Q1	REF			REF			REF		
	Q2	0.80 (0.38–1.69)	0.79 (0.40–1.58)	0.79 (0.40–1.58)	0.86 (0.53–1.40)	0.90 (0.55–1.46)	0.89 (0.55–1.44)	0.75 (0.28–2.01)	0.72 (0.27–1.88)	0.73 (0.30–1.79)
	Q3	0.88 (0.36–2.14)	0.89 (0.41–1.92)	0.88 (0.41–1.91)	0.70 (0.36–1.36)	0.69 (0.36–1.34)	0.66 (0.34–1.29)	1.01 (0.32–3.14)	0.98 (0.34–2.85)	1.01 (0.39–2.61)
	Q4	1.23 (0.68–2.23)	1.13 (0.66–1.95)	1.13 (0.66–1.94)	1.13 (0.66–1.91)	1.08 (0.64–1.81)	1.07 (0.62–1.85)	1.29 (0.66–2.53)	1.20 (0.62–2.34)	1.14 (0.63–2.05)
Hyperuricemia	Q1	REF			REF			REF		
	Q2	1.04 (0.53–2.02)	1.09 (0.57–2.09)	1.09 (0.57–2.09)	1.09 (0.43–2.81)	1.16 (0.49–2.72)	1.15 (0.49–2.69)	0.92 (0.57–1.46)	1.00 (0.64–1.54)	0.99 (0.64–1.53)
	Q3	1.02 (0.72–1.43)	1.07 (0.74–1.55)	1.08 (0.74–1.56)	0.89 (0.62–1.28)	0.97 (0.69–1.37)	0.96 (0.67–1.39)	1.09 (0.72–1.67)	1.20 (0.78–1.86)	1.21 (0.78–1.87)
	Q4	0.71 (0.56–0.92) *	0.74 (0.58–0.95) *	0.74 (0.58–0.95) *	0.74 (0.58–0.94) *	0.80 (0.64–0.99) *	0.79 (0.64–0.98) *	0.71 (0.46–1.08)	0.71 (0.51–1.00) *	0.72 (0.51–1.01)

Q: quartile; CI: confidence interval; OR: odds ratio; CVD: cardiovascular diseases; REF: reference category. * Boldface indicates statistical significance (*p* < 0.05). ** The Russian deprivation index measures general deprivation, and its components measure social, economic, and environmental deprivation, respectively. *** Q1—the least deprived areas; Q4—the most deprived areas. Model 0 was unadjusted; Model 1 was adjusted for independent variables that the random forest algorithm identified as “important”; Model 2—Model 0 + age, sex (for total population), marital status, income and education level, smoking and alcohol drinking status, place of residence, and dietary habits (consumption of sugar, salt, dairy fats, vegetables, and fruit).

**Table 3:** Association of economic deprivation ** with CVD risk factors.

Dependent Variables	Level of Deprivation ***	Total Population			Men			Women		
		Model 0	Model 1	Model 2	Model 0	Model 1	Model 2	Model 0	Model 1	Model 2
		OR (95% CI)	OR (95% CI)	OR (95% CI)	OR (95% CI)	OR (95% CI)	OR (95% CI)	OR (95% CI)	OR (95% CI)	OR (95% CI)
Hypertension	Q1	REF			REF			REF		
	Q2	1.52 (1.00–2.30)	1.54 (1.09–2.18) *	1.55 (1.10–2.18) *	1.52 (0.92–2.52)	1.63 (1.04–2.55) *	1.62 (1.03–2.55) *	1.51 (0.98–2.33)	1.48 (1.09–2.00) *	1.49 (1.10–2.00) *
	Q3	1.40 (0.99–2.00)	1.62 (1.20–2.19) *	1.62 (1.20–2.19) *	1.61 (1.01–2.56) *	1.78 (1.18–2.68) *	1.76 (1.14–2.72) *	1.28 (0.91–1.80)	1.53 (1.22–1.93) *	1.54 (1.22–1.94) *
	Q4	2.99 (2.12–4.23) *	2.83 (2.09–3.82) *	2.82 (2.10–3.79) *	2.72 (1.73–4.26) *	2.80 (1.89–4.13) *	2.90 (1.91–4.40) *	3.17 (2.33–4.31) *	2.78 (2.22–3.47) *	2.77 (2.22–3.45) *
Elevated blood pressure	Q1	REF			REF			REF		
	Q2	1.43 (1.06–1.92) *	1.41 (0.98–2.03)	1.42 (0.98–2.07)	1.36 (1.01–1.84) *	1.32 (0.94–1.87)	1.33 (0.93–1.91)	1.49 (1.04–2.12) *	1.48 (0.99–2.20)	1.47 (0.98–2.21)
	Q3	1.78 (1.40–2.27) *	1.70 (1.27–2.27) *	1.70 (1.26–2.28) *	1.56 (1.17–2.08) *	1.50 (1.09–2.05) *	1.50 (1.13–2.00) *	1.93 (1.44–2.60) *	1.87 (1.35–2.59) *	1.84 (1.32–2.57) *
	Q4	0.51 (0.43–0.60) *	0.53 (0.41–0.68) *	0.53 (0.41–0.68) *	0.37 (0.30–0.44) *	0.38 (0.29–0.48) *	0.37 (0.30–0.47) *	0.65 (0.50–0.83) *	0.68 (0.51–0.91) *	0.68 (0.50–0.92) *
Obesity	Q1	REF			REF			REF		
	Q2	1.17 (0.75–1.82)	1.11 (0.78–1.57)	1.55 (1.10–2.18) *	1.04 (0.71–1.51)	-	1.03 (0.74–1.45)	1.24 (0.77–1.98)	1.13 (0.79–1.61)	1.14 (0.79–1.64)
	Q3	1.06 (0.71–1.59)	1.12 (0.82–1.52)	1.62 (1.20–2.19) *	1.10 (0.77–1.57)	-	1.09 (0.78–1.51)	1.06 (0.70–1.61)	1.11 (0.81–1.52)	1.13 (0.83–1.54)
	Q4	1.83 (1.23–2.71) *	1.61 (1.20–2.16) *	2.82 (2.10–3.79) *	1.45 (1.03–2.04) *	-	1.42 (1.05–1.92) *	2.08 (1.39–3.11) *	1.71 (1.28–2.30) *	1.71 (1.28–2.28) *
Diabetes mellitus	Q1	REF			REF			REF		
	Q2	1.01 (0.73–1.40)	1.04 (0.79–1.36)	1.05 (0.80–1.37)	0.81 (0.48–1.37)	0.88 (0.62–1.24)	0.86 (0.63–1.17)	1.15 (0.86–1.53)	1.19 (0.84–1.68)	1.18 (0.85–1.65)
	Q3	0.98 (0.75–1.28)	1.16 (1.00–1.35) *	1.14 (0.97–1.36)	0.86 (0.52–1.43)	1.01 (0.75–1.36)	0.97 (0.73–1.29)	1.07 (0.84–1.36)	1.30 (1.03–1.64) *	1.29 (1.00–1.66)
	Q4	2.08 (1.73–2.50) *	1.80 (1.71–1.89) *	1.80 (1.70–1.90) *	1.62 (1.02–2.57) *	1.63 (1.27–2.08) *	1.61 (1.28–2.04) *	2.42 (2.37–2.46) *	1.95 (1.69–2.25) *	1.92 (1.67–2.19) *
Prediabetes	Q1	REF			REF			REF		
	Q2	1.96 (1.18–3.26) *	1.89 (1.18–3.04) *	1.89 (1.16–3.07) *	1.49 (0.76–2.93)	1.46 (0.75–2.84)	1.45 (0.77–2.72)	2.57 (1.29–5.13) *	2.39 (1.29–4.44) *	2.41 (1.31–4.44) *
	Q3	2.62 (1.68–4.09) *	2.66 (1.73–4.08) *	2.64 (1.71–4.10) *	2.08 (1.32–3.29) *	2.10 (1.35–3.24) *	2.13 (1.39–3.28) *	3.21 (1.64–6.28) *	3.23 (1.72–6.06) *	3.24 (1.73–6.05) *
	Q4	5.96 (3.95–8.99) *	5.49 (3.73–8.09) *	5.51 (3.74–8.13) *	4.49 (3.02–6.68) *	4.18 (2.90–6.02) *	4.27 (3.01–6.06) *	7.93 (4.12–15.25) *	6.89 (3.79–12.53) *	6.90 (3.80–12.52) *
Chronic kidney disease	Q1	REF			REF			REF		
	Q2	2.20 (1.08–4.49) *	2.19 (1.22–3.92) *	2.18 (1.23–3.84) *	1.68 (1.17–2.41) *	1.66 (1.16–2.36) *	1.60 (1.07–2.40) *	2.52 (0.93–6.78)	2.51 (1.04–6.06) *	2.47 (1.11–5.51) *
	Q3	1.31 (0.68–2.53)	1.40 (0.81–2.41)	1.40 (0.81–2.40)	1.36 (0.83–2.23)	1.35 (0.80–2.29)	1.38 (0.83–2.30)	1.28 (0.54–3.01)	1.33 (0.60–2.93)	1.38 (0.70–2.73)
	Q4	2.51 (1.44–4.37) *	2.32 (1.56–3.44) *	2.31 (1.56–3.42) *	2.01 (1.60–2.52) *	1.97 (1.47–2.65) *	1.90 (1.48–2.45) *	2.80 (1.27–6.20) *	2.83 (1.41–5.68) *	2.55 (1.41–4.60) *
Hyperuricemia	Q1	REF			REF			REF		
	Q2	1.24 (0.98–1.56)	1.27 (1.04–1.54) *	1.27 (1.04–1.55) *	1.06 (0.72- 1.55)	1.11 (0.80–1.53)	1.10 (0.80–1.53)	1.41 (1.09–1.82) *	1.38 (1.07–1.77) *	1.39 (1.09–1.77) *
	Q3	2.00 (1.47–2.74) *	2.07 (1.52–2.84) *	2.07 (1.51–2.85) *	1.86 (1.16–2.98) *	1.84 (1.16–2.91) *	1.87 (1.18–2.94) *	2.06 (1.45–2.92) *	2.24 (1.49–3.37) *	2.24 (1.49–3.36) *
	Q4	1.60 (1.28–2.01) *	1.57 (1.31–1.88) *	1.56 (1.30–1.88) *	1.16 (0.80–1.68)	1.20 (0.87–1.65)	1.22 (0.89–1.67)	2.05 (1.63–2.57) *	1.77 (1.40–2.25) *	1.78 (1.41–2.25) *

Q: quartile; CI: confidence interval; OR: odds ratio; CVD: cardiovascular diseases; REF: reference category. * Boldface indicates statistical significance (*p* < 0.05). ** The Russian deprivation index measures general deprivation, and its components measure social, economic, and environmental deprivation, respectively. *** Q1—the least deprived areas; Q4—the most deprived areas. Model 0 was unadjusted; Model 1 was adjusted for independent variables that the random forest algorithm identified as “important”; Model 2—Model 0 + age, sex (for total population), marital status, income and education level, smoking and alcohol drinking status, place of residence, and dietary habits (consumption of sugar, salt, dairy fats, vegetables, and fruit).

**Table 4:** Association of environmental deprivation ** with CVD risk factors.

Dependent Variables	Level of Deprivation ***	Total Population			Men			Women		
		Model 0	Model 1	Model 2	Model 0	Model 1	Model 2	Model 0	Model 1	Model 2
		OR (95% CI)	OR (95% CI)	OR (95% CI)	OR (95% CI)	OR (95% CI)	OR (95% CI)	OR (95% CI)	OR (95% CI)	OR (95% CI)
Hypertension	Q1	REF			REF			REF		
	Q2	1.27 (1.10–1.47) *	1.46 (1.35–1.58) *	1.47 (1.35–1.59) *	0.99 (0.97–1.01)	1.24 (1.14–1.35) *	1.17 (1.10–1.24) *	1.47 (1.19–1.81) *	1.64 (1.43–1.88) *	1.65 (1.42–1.90) *
	Q3	1.13 (0.66–1.95)	1.17 (0.71–1.90)	1.17 (0.72–1.91)	1.00 (0.57–1.76)	1.09 (0.64–1.86)	1.05 (0.61–1.81)	1.21 (0.69–2.11)	1.25 (0.78–1.98)	1.25 (0.79–1.99)
	Q4	1.21 (0.98–1.50)	1.37 (1.19–1.57) *	1.37 (1.20–1.56) *	1.23 (1.06–1.42) *	1.38 (1.19–1.61) *	1.36 (1.20–1.53) *	1.18 (0.86–1.63)	1.37 (1.14–1.65) *	1.37 (1.14–1.65) *
Elevated blood pressure	Q1	REF			REF			REF		
	Q2	2.15 (1.84–2.52) *	2.00 (1.59–2.53) *	1.99 (1.57–2.53) *	2.13 (1.88–2.42) *	1.89 (1.49–2.40) *	1.93 (1.52–2.45) *	2.10 (1.68–2.63) *	2.01 (1.56–2.60) *	1.97 (1.50–2.61) *
	Q3	0.92 (0.56–1.51)	0.86 (0.52–1.44)	0.86 (0.51–1.45)	0.77 (0.50–1.19)	0.72 (0.46–1.15)	0.73 (0.46–1.16)	1.01 (0.57–1.80)	0.99 (0.56–1.76)	0.99 (0.56–1.77)
	Q4	1.50 (1.24–1.80) *	1.39 (1.08–1.78) *	1.39 (1.07–1.80) *	1.31 (1.09–1.57) *	1.22 (0.94–1.59)	1.26 (0.98–1.62)	1.57 (1.22–2.00) *	1.49 (1.13–1.97) *	1.47 (1.10–1.98) *
Obesity	Q1	REF			REF			REF		
	Q2	1.04 (0.73–1.49)	1.09 (0.83–1.44)	1.09 (0.83–1.44)	1.01 (0.79–1.28)	1.06 (0.77–1.45)	1.06 (0.77–1.45)	1.08 (0.73–1.60)	1.05 (0.79–1.40)	1.08 (0.80–1.44)
	Q3	0.90 (0.54–1.51)	0.91 (0.60–1.39)	0.91 (0.60–1.39)	0.82 (0.56–1.20)	0.84 (0.56–1.26)	0.84 (0.56–1.26)	0.98 (0.55–1.73)	0.95 (0.61–1.48)	0.95 (0.61–1.48)
	Q4	0.96 (0.66–1.41)	1.03 (0.77–1.38)	1.03 (0.77–1.38)	0.91 (0.70–1.18)	0.92 (0.67–1.27)	0.93 (0.67–1.27)	1.03 (0.67–1.59)	1.07 (0.79–1.47)	1.09 (0.79–1.49)
Diabetes mellitus	Q1	REF			REF			REF		
	Q2	0.85 (0.70–1.03)	1.12 (1.05–1.20) *	1.12 (1.05–1.20) *	0.57 (0.36–0.91) *	0.85 (0.63–1.15)	0.84 (0.62–1.14)	1.06 (1.03–1.09) *	1.31 (1.20–1.43) *	1.28 (1.15–1.41) *
	Q3	1.34 (0.93–1.93)	1.53 (1.30–1.81) *	1.53 (1.30–1.81) *	0.88 (0.50–1.57)	1.09 (0.76–1.57)	1.07 (0.75–1.52)	1.70 (1.28–2.27) *	1.89 (1.62–2.21) *	1.86 (1.59–2.18) *
	Q4	0.83 (0.66–1.06)	1.00 (0.85–1.17)	0.99 (0.86–1.15)	0.68 (0.41–1.14)	0.85 (0.59–1.22)	0.83 (0.58–1.17)	0.93 (0.81–1.07)	1.12 (0.97–1.29)	1.10 (0.95–1.28)
Prediabetes	Q1	REF			REF			REF		
	Q2	2.17 (1.36–3.47) *	2.18 (1.43–3.34) *	2.16 (1.40–3.32) *	1.50 (1.34–1.68) *	1.57 (1.41–1.77) *	1.59 (1.42–1.77) *	2.95 (1.29–6.71) *	2.80 (1.29–6.06) *	2.81 (1.32–6.01) *
	Q3	2.17 (0.92–5.13)	2.15 (0.97–4.76)	2.14 (0.96–4.76)	1.43 (0.69–2.95)	1.45 (0.75–2.80)	1.49 (0.77–2.89)	3.02 (1.00–9.14)	2.96 (1.06–8.26) *	2.98 (1.08–8.21) *
	Q4	2.11 (1.25–3.55) *	2.08 (1.30–3.34) *	2.07 (1.28–3.33) *	1.49 (1.03–2.16) *	1.50 (1.03–2.20) *	1.53 (1.06–2.21) *	2.72 (1.17–6.33) *	2.69 (1.23–5.92) *	2.71 (1.25–5.86) *
Chronic kidney disease	Q1	REF			REF			REF		
	Q2	1.62 (0.84–3.12)	1.74 (1.04–2.90) *	1.73 (1.04–2.88) *	2.10 (1.48–3.00) *	2.26 (1.45–3.53) *	2.39 (1.60–3.59) *	1.41 (0.66–3.03)	1.45 (0.77–2.74)	1.50 (0.85–2.63)
	Q3	1.89 (0.83–4.29)	1.93 (0.99–3.73)	1.92 (0.99–3.71)	1.71 (1.09–2.68) *	1.87 (1.17–2.99) *	1.82 (1.19–2.76) *	2.01 (0.72–5.60)	2.01 (0.83–4.90)	2.03 (0.91–4.53)
	Q4	1.25 (0.58–2.71)	1.33 (0.71–2.49)	1.32 (0.71–2.44)	1.42 (0.83–2.42)	1.48 (0.85–2.58)	1.49 (0.88–2.53)	1.19 (0.49–2.88)	1.22 (0.56–2.65)	1.25 (0.65–2.39)
Hyperuricemia	Q1	REF			REF			REF		
	Q2	1.68 (1.28–2.20) *	1.75 (1.40–2.18) *	1.74 (1.40–2.17) *	1.51 (1.26–1.81) *	1.46 (1.33–1.61) *	1.49 (1.33–1.66) *	1.77 (1.30–2.41) *	1.83 (1.35–2.50) *	1.84 (1.36–2.50) *
	Q3	1.15 (0.80–1.65)	1.18 (0.87–1.59)	1.18 (0.88–1.59)	0.73 (0.54–0.98) *	0.78 (0.63–0.96) *	0.78 (0.63–0.98) *	1.56 (1.05–2.32) *	1.56 (1.08–2.25) *	1.57 (1.09–2.25) *
	Q4	1.64 (1.12–2.41) *	1.74 (1.22–2.48) *	1.74 (1.22–2.48) *	1.32 (0.91–1.90)	1.37 (0.97–1.93)	1.40 (0.99–1.97)	1.84 (1.20–2.82) *	1.94 (1.22–3.08) *	1.95 (1.24–3.09) *

Q: quartile; CI: confidence interval; OR: odds ratio; CVD: cardiovascular diseases; REF: reference category. * Boldface indicates statistical significance (*p* < 0.05). ** The Russian deprivation index measures general deprivation, and its components measure social, economic, and environmental deprivation, respectively. *** Q1—the least deprived region; Q4—the most deprived region. Model 0 was unadjusted; Model 1 was adjusted for independent variables that the random forest algorithm identified as “important”; Model 2—Model 0 + age, sex (for total population), marital status, income and education level, smoking and alcohol drinking status, place of residence, and dietary habits (consumption of sugar, salt, dairy fats, vegetables, and fruit).

## Data Availability

The raw data supporting the conclusions of this article will be made available by the authors on request.

## Supplementary Materials

The following supporting information can be downloaded at: https://www.mdpi.com/article/10.3390/ijerph22040594/s1. Table S1: Components of the Russian deprivation index; Figure S1: The federal subjects of Russia, stratified by level of general deprivation; Figure S2: The federal subjects of Russia, stratified by level of social deprivation; Figure S3: The federal subjects of Russia, stratified by level of economic deprivation; Figure S4: The federal subjects of Russia, stratified by level of environmental deprivation; Table S2: Definitions of deprivation indicators; Table S3: Assessment of the importance of independent variables in predicting the value of the target variable, using the random forest algorithm, in the total population; Table S4: Assessment of the importance of independent variables in predicting the value of the target variable, using the random forest algorithm, in women; Table S5: Assessment of the importance of independent variables in predicting the value of the target variable, using the random forest algorithm, in men; Table S6: Baseline characteristics of study participants by quartile of general deprivation; Table S7: Baseline characteristics of study participants by quartile of social deprivation; Table S8: Baseline characteristics of study participants by quartile of economic deprivation; Table S9: Baseline characteristics of study participants by quartile of environmental deprivation; Table S10: Association of general deprivation with baseline blood pressure (SBP and DBP), creatinine, fasting glucose, uric acid levels, and body mass index; Table S11: Association of social deprivation with baseline blood pressure (SBP and DBP), creatinine, fasting glucose, uric acid levels, and body mass index; Table S12: Association of economic deprivation with baseline blood pressure (SBP and DBP), creatinine, fasting glucose, uric acid levels, and body mass index; Table S13: Association of environmental deprivation with baseline blood pressure (SBP and DBP), creatinine, fasting glucose, uric acid levels, and body mass index; Table S14: R code for total population.; Table S15: R code for men; Table S16: R code for women; Table S17: R code for total population, men and women.

## Abbreviations

The following abbreviations are used in this manuscript:

CVD	Cardiovascular diseases
HTN	Hypertension
DM	Diabetes mellitus
HUA	Hyperuricemia
ESSE-RF	The Epidemiology of Cardiovascular Risk Factors and Diseases in Regions of the Russian Federation
SBP	Systolic blood pressure
DBP	Diastolic blood pressure
CKD	Chronic kidney disease
SUA	Serum uric acid
BMI	Body mass index
OR	Odds ratio
GFR	Glomerular filtration rate
GDP	Gross domestic product
RR	Rate ratio
HR	Hazard ratio
CI	Confidence interval
Q	Quartile
RDI	Russian deprivation index
