# Supplementary material for: Association Between Area-Level Deprivation and Cardio-Metabolic Risk Factors Among the Adult Population in Russia
Source: Int J Environ Res Public Health. 2025 Apr 10;22(4):594. doi: 10.3390/ijerph22040594 (PMC12026931; doi:10.3390/ijerph22040594)
Supplement: Supplementary file 1 [file ijerph-22-00594-s001.zip › ijerph-3463479-supplementary.pdf]

Contents

Table S1: Components of Russian deprivation index. 2

Figure S1: The federal subjects of Russia stratified by level of general deprivation. 4

Figure S2: The federal subjects of Russia stratified by level of social deprivation. 4

Figure S3: The federal subjects of Russia stratified by level of economic deprivation. 5

Figure S4: The federal subjects of Russia stratified by level of environmental deprivation. 5

Table S2: Definitions of deprivation indicators. 6

Table S3: Assessment of the importance of independent variables in predicting the value of the target variable using the random forest algorithm in total population. 7

Table S4: Assessment of the importance of independent variables in predicting the value of the target variable using the random forest algorithm in women. 8

Table S5: Assessment of the importance of independent variables in predicting the value of the target variable using the random forest algorithm in men. 10

Table S6: Baseline characteristics of study participants by quartile of general deprivation. 11

Table S7: Baseline characteristics of study participants by quartile of social deprivation. 19

Table S8: Baseline characteristics of study participants by quartile of economic deprivation. 24

Table S9: Baseline characteristics of study participants by quartile of environmental deprivation. 30

Table 10: Association of general deprivation with baseline blood pressure (SBP and DBP), creatinine, fasting glucose, uric acid levels and body mass index. 36

Table S11: Association of social deprivation with baseline blood pressure (SBP and DBP), creatinine, fasting glucose, uric acid levels and body mass index. 37

Table S12: Association of economic deprivation with baseline blood pressure (SBP and DBP), creatinine, fasting glucose, uric acid levels and body mass index. 39

Table S13: Association of environmental deprivation with baseline blood pressure (SBP and DBP), creatinine, fasting glucose, uric acid levels and body mass index. 41

Table S14: R code for total population. 43

Table S15: R code for men. 45

Table S16: R code for women. 47

Table S17: R code for total population, men and women. 48

Table S1: Components of the Russian deprivation index.

Social deprivation	Economic deprivation	Environmental deprivation
Living in crowded households	Stove heating	Dead forest
Families with children under age 5 years old	No hot water supply	Fire forest incidence
Families with 3 and more children (aged 0–18)	No sewerage system	Environmental crime
Unemployment rate	Not central sewerage system	Transport-related emissions
Households with phone	Low income	Emissions from stationary sources: NO_2, SO_2, CO

The federal subjects of Russia in 2010

These are the federal subjects, of which there are 83 in Russia. The 83 federal subjects comprise various different types of unit. There are 46 regions, 21 Republics, 9 territories, 4 autonomous areas, 2 cities of federal importance and 1 autonomous region. The federal districts are groupings of the federal subjects of Russia. Russia is divided into eight federal districts. The federal districts are not an additional or higher level of federal structure but are responsible for organizing the federal government's activities in different federal divisions. The Central Federal District is one of the 8 federal districts of Russia. Its surface area is 650,200 square kilometers and its population is 38,427,539 according to the 2010 census. The Central Federal District consists of 18 federal units. Geographically, the region is located in the most western part of present-day Russia. The Northwestern Federal District is made up of 11 federal units. The region is located north of the European part of Russia. Its surface area is 1,687,000 square kilometers and its population is 13,616,057 according to the 2010 census. The South Federal District located in the south of European Russia and consists of 6 federal units. Its surface area is 4,209,000 square kilometers and its population is 13,854,334 according to the 2010 census. The North Caucasian Federal District is made up of 7 federal units. The region is located in the northern part of the Caucasus and is located in the southwestern region of Russia. Its surface area is 170,400 square kilometers and its population is 9,428,826 according to the 2010 census. The Volga Federal District is made up of 14 federal units. The region is the southeastern part of Russia's European region. Its surface area is 1,037,000 square kilometers and its population is 29,899,699 according to the 2010 census. The Ural Federal District is located on the border of Russia's European and Asian regions. The Ural Federal District consists of 6 federal units. Its surface area is 1,818,500 km^2 and its population is 12,080,526 according to 2010 census. The Siberian Federal District is located in the north of Central Asia and consists of 12 federal units. Its surface area is 5,145,000 km^2 and its population is 19,256,426 according to 2010 census. The Far Eastern Federal District covers the Far East region of Russia and consists of 9 federal units. Its surface area is 6,169,300 km^2 and its population is 6,293,129 according to the 2010 census.

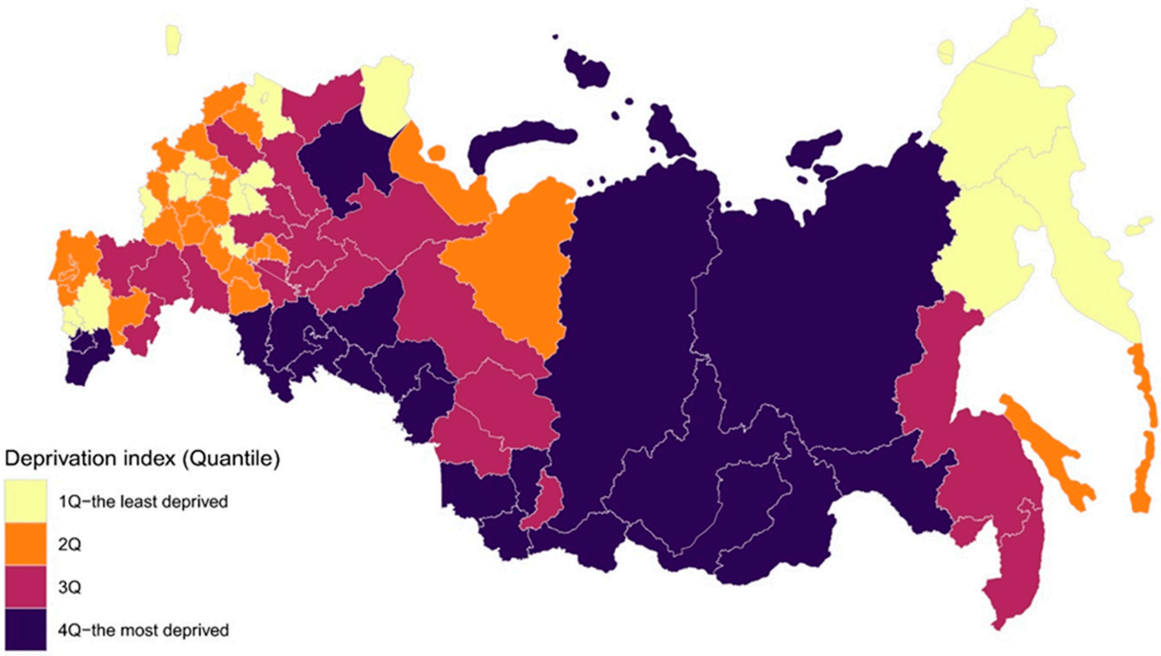

Figure S1: The federal subjects of Russia stratified by level of general deprivation.

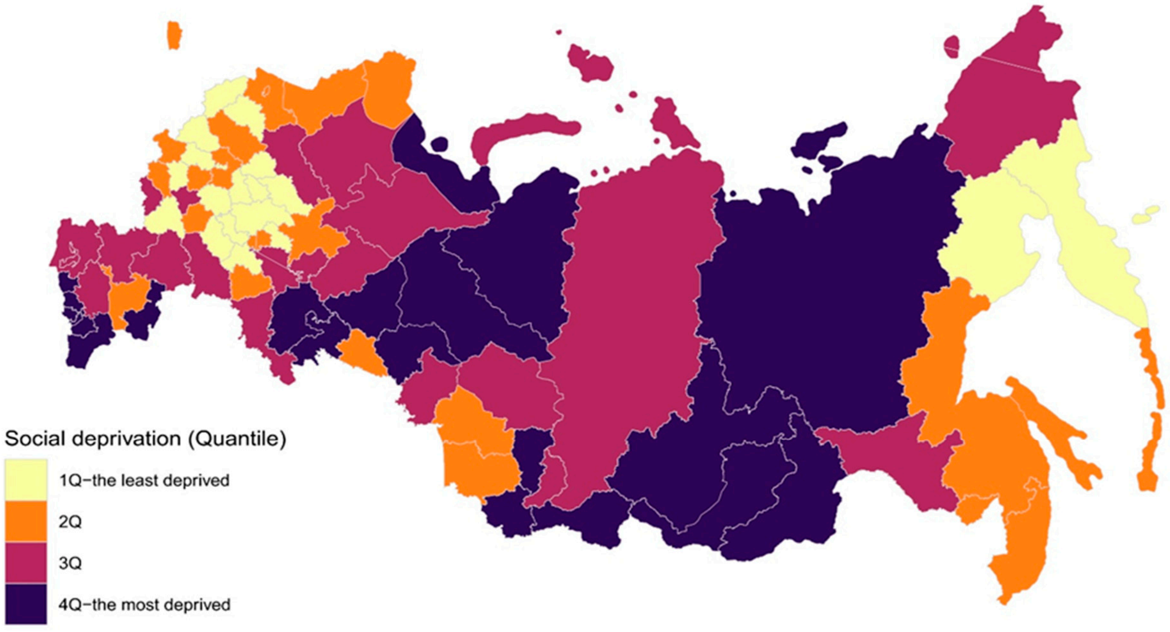

Figure S2: The federal subjects of Russia stratified by level of social deprivation.

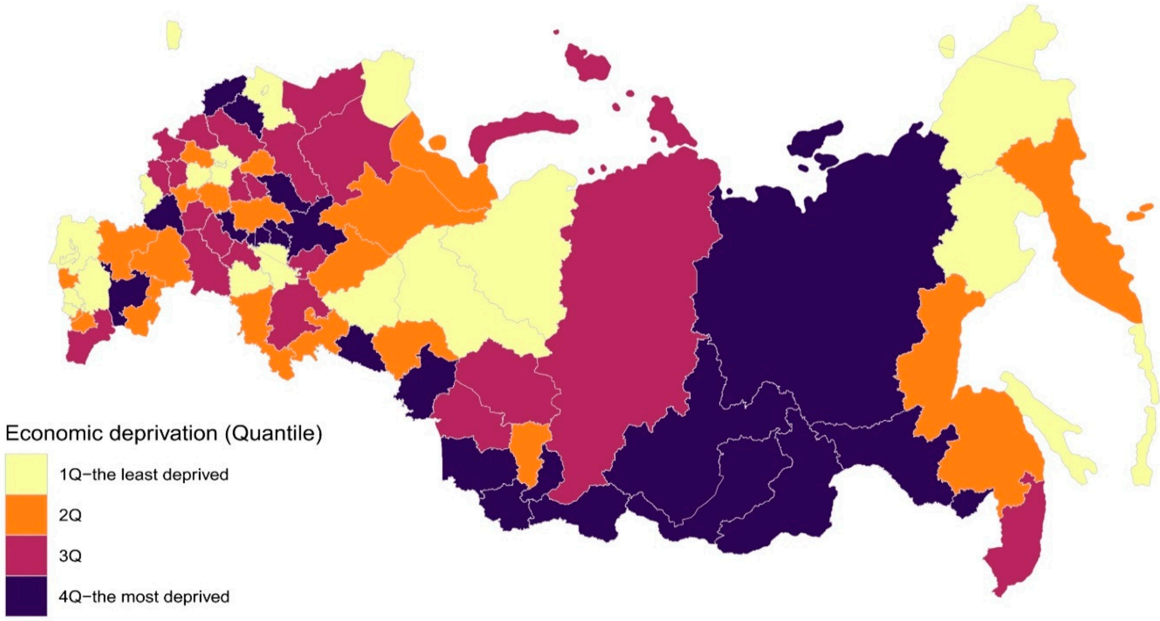

Figure S3: The federal subjects of Russia stratified by level of economic deprivation.

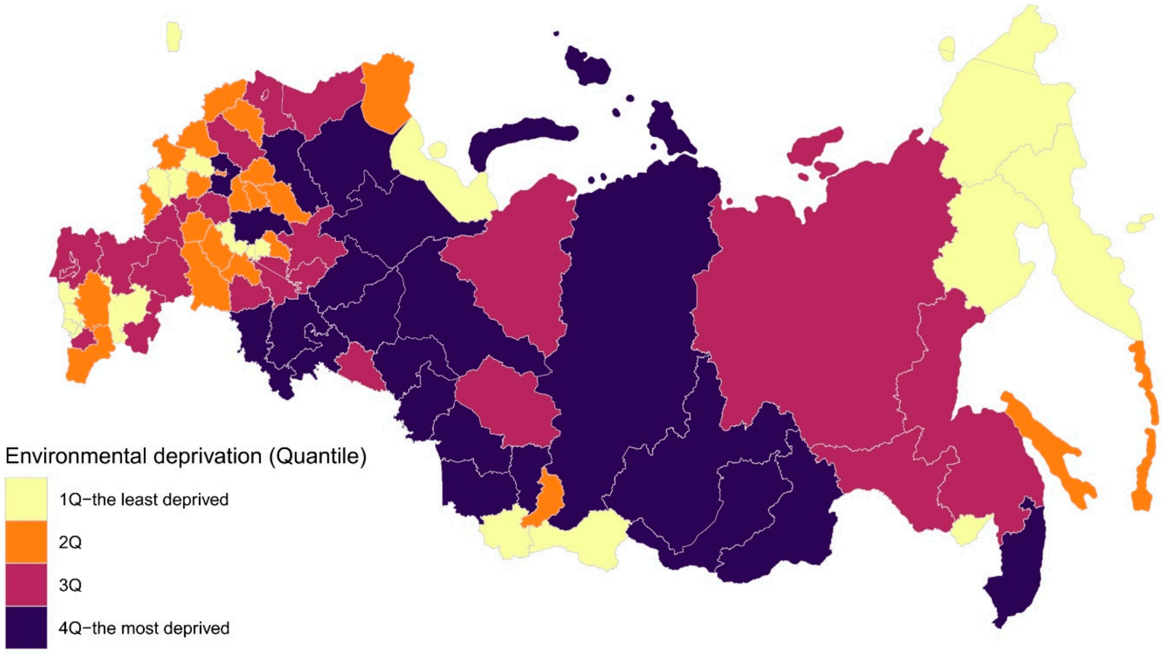

Figure S4: The federal subjects of Russia stratified by level of environmental deprivation.

Table S2: Definitions of deprivation indicators.

Domain	Variable	Description	Data source
Family structure /	Children +3	Percentage of families with 3 and more children (ages 0-18)	Census 2010
Demographics	Children under 5 years old	Children ages 0-4 as a percentage of total population	Census 2010
Housing	Stove heating	Percentage of households with stove heating	Census 2010
	No hot water supply	Percentage of households without heat water supply	Census 2010
	No central sewerage system	Percentage of households with toilets emptying into a cesspit	Census 2010
	No sewerage system	Percentage of households without sewage system	Census 2010
	Overcrowded	Percentage of households (individual (single-family) houses, individual and communal apartments) with > 5 persons	Census 2010
Communicatio n	Phone	Percentage of households with telephone	Census 2010
Income and Wealth	Low income	Percentage of people below a low income threshold in the total population	Regions of Russia. Social and Economic Indicators – 2011
	Unemploymen t rate	Population 15 or older unemployed	Labour and Employment in Russia – 2011
Air quality	NO_2	Nitrogen dioxide (thousand tons) from stationary sources	Environment Protection in Russia – 2012
	SO_2	Sulphur dioxide (thousand tons) from stationary sources	Environment Protection in Russia – 2012
	CO	Carbon monoxide (thousand tons) from stationary sources	Environment Protection in Russia – 2012
	Transport-related emissions	Air emissions from vehicle (thousand tons)	Environment Protection in Russia – 2012
Natural disaster	Fire forest incidence	The number of fire forest incidence (unit)	Environment Protection in Russia – 2012
Green space	Area of dead forest	The area of dead forest (hectares)	Environment Protection in Russia – 2012
Crimes	Environmental crimes	The number of recorded environmental crimes	Environment Protection in Russia – 2012

Table S3: Assessment of the importance of independent variables in predicting the value of the target variable using the random forest algorithm in total population.

Independent variables	Target variables						
	Elevated blood pressure	Hypertension	Prediabetes	Diabetes mellitus	Hyperuricemia	Obesity	Chronic kidney disease
Age	+	+	+	+	+	+	*
Sex	+	+	+	+	*	+	*
Income	+	+	+	+	+	+	+
Achieved education	+	+	+		+	+	+
				+			
Marital status	+	+	+	+		+	+
Smoking status	+	+	+	+	+	+	+
Alcohol drinking status	+	+		+	+	+	+
Place of residence	+	+	+		+	+	+
High level of sugar consumption		+	+	+	+	+	+
High level of salt consumption		+			+		
High level of dairy fat consumption			+				
Low levels of fruit and vegetables consumption		+		+	+	+	+

Independent variables	Target variables						
	Elevated blood pressure	Hypertension	Prediabetes	Diabetes mellitus	Hyperuricemia	Obesity	Chronic kidney disease
General deprivation	+	+	+	+	+	+	+
Social deprivation	+	+	+	+	+	+	+
Economic deprivation	+	+	+	+	+	+	+
Environmental deprivation	+	+	+	+	+	+	+

+ – important variables; *variables not included in the random forest algorithm.

Table S4: Assessment of the importance of independent variables in predicting the value of the target variable using the random forest algorithm in women.

Independent variables	Target variables						
	Elevated blood pressure	Hypertension	Prediabetes	Diabetes mellitus	Hyperuricemia	Obesity	Chronic kidney disease
Age	+	+	+	+	+	+	*
Income	+	+	+	+	+	+	+
Achieved education	+	+	+		+	+	
Marital status	+	+	+	+	+	+	
Smoking status	+	+	+	+	+	+	+
Alcohol drinking status	+	+	+	+	+	+	
Place of residence	+	+	+		+	+	+

Independent variables	Target variables						
	Elevated blood pressure	Hypertension	Prediabetes	Diabetes mellitus	Hyperuricemia	Obesity	Chronic kidney disease
High level of sugar consumption		+	+	+	+		+
High level of salt consumption		+					
High level of dairy fat consumption							
Low levels of fruit and vegetables consumption				+			
General deprivation	+	+	+	+	+	+	+
Social deprivation	+	+	+	+	+	+	+
Economic deprivation	+	+	+	+	+	+	+
Environmental deprivation	+	+	+	+	+	+	+

+ – important variables; *variables not included in the random forest algorithm.

Table S5: Assessment of the importance of independent variables in predicting the value of the target variable using the random forest algorithm in men.

Independent variables	Target variables						
	Elevated blood pressure	Hypertension	Prediabetes	Diabetes mellitus	Hyperuricemia	Obesity	Chronic kidney disease
Age	+	+	+	+	+	+	*
Income			+	+	+		+
Achieved education		+	+	+	+	+	+
Marital status	+	+	+	+	+	+	
Smoking status		+			+	+	+
Alcohol drinking status	+			+	+	+	+
Place of residence		+			+	+	+
High level of sugar consumption				+		+	
High level of salt consumption							
High level of dairy fat consumption							
Low levels of fruit and vegetables consumption				+		+	+
General deprivation	+	+	+	+	+	+	+
Social deprivation	+	+	+	+	+	+	+
Economic deprivation	+	+	+	+	+		+

Independent variables	Target variables						
	Elevated blood pressure	Hypertension	Prediabetes	Diabetes mellitus	Hyperuricemia	Obesity	Chronic kidney disease
Environmental deprivation	+	+	+	+	+	+	+

+ – important variables; *variables not included in the random forest algorithm.

Table S6: Baseline characteristics of study participants by quartile* of general deprivation.

Characteristic	General deprivation**				p value[a]
	Q1 (N=3459)	Q2 (N=3173)	Q3 (N=5324)	Q4 (N=9965)	
Place of residence, N (%)					<0.0001
Urban	3134 (90.60%)	2776 (87.49%)	3930 (73.82%)	7888 (79.16%)	
Rural	325 (9.40%)	397 (12.51%)	1394 (26.18%)	2077 (20.84%)	
Sex, N (%)					<0.0001
Men	1253 (36.22%)	1278 (40.28%)	2280 (42.82%)	3564 (35.77%)	
Women	2206 (63.78%)	1895 (59.72%)	3044 (57.18%)	6401 (64.23%)	

Characteristic	General deprivation**				p value[a]
	Q1 (N=3459)	Q2 (N=3173)	Q3 (N=5324)	Q4 (N=9965)	
Age (years), Med [IQR]	48.0 [37.0;56.0]	49.0 [37.0;57.0]	47.0 [35.0;55.0]	49.0 [37.0;57.0]	<0.0001
Marital status, N (%)					<0.0001
Single	493 (14.25%)	485 (15.29%)	806 (15.14%)	1520 (15.25%)	
Married	2257 (65.25%)	2012 (63.41%)	3484 (65.44%)	6406 (64.28%)	
Windowed	496 (14.34%)	389 (12.26%)	722 (13.56%)	1269 (12.73%)	
Divorced	213 (6.16%)	287 (9.05%)	312 (5.86%)	770 (7.73%)	
Income, N (%)					<0.0001
Low income	353 (10.21%)	449 (14.15%)	629 (11.81%)	1575 (15.81%)	
Average income	2381 (68.83%)	2446 (77.09%)	3748 (70.40%)	7366 (73.92%)	
Higher income	725 (20.96%)	278 (8.76%)	947 (17.79%)	1024 (10.28%)	

Characteristic	General deprivation**				p value[a]
	Q1 (N=3459)	Q2 (N=3173)	Q3 (N=5324)	Q4 (N=9965)	
Achieved education, N (%)					<0.0001
Primary level	409 (11.82%)	497 (15.66%)	728 (13.67%)	2024 (20.31%)	
Secondary level	1227 (35.47%)	1280 (40.34%)	1825 (34.28%)	3580 (35.93%)	
Higher level	1823 (52.70%)	1396 (44.00%)	2771 (52.05%)	4361 (43.76%)	
Smoking status, N (%)					<0.0001
Never	1986 (57.42%)	2097 (66.09%)	2781 (52.24%)	6453 (64.76%)	
Former	734 (21.22%)	423 (13.33%)	1237 (23.23%)	1383 (13.88%)	
Current	739 (21.36%)	653 (20.58%)	1306 (24.53%)	2129 (21.36%)	
Alcohol drinking status, N (%)					<0.0001
Never	462 (13.36%)	758 (23.89%)	1047 (19.67%)	3079 (30.90%)	
Moderate	2822 (81.58%)	2344 (73.87%)	4090 (76.82%)	6614 (66.37%)	

Characteristic	General deprivation**				p value[a]
	Q1 (N=3459)	Q2 (N=3173)	Q3 (N=5324)	Q4 (N=9965)	
Hard–drinking	175 (5.06%)	71 (2.24%)	187 (3.51%)	272 (2.73%)	
High level of sugar consumption, N (%)					<0.0001
No	1639 (47.38%)	1669 (52.60%)	2740 (51.47%)	5232 (52.50%)	
Yes	1820 (52.62%)	1504 (47.40%)	2584 (48.53%)	4733 (47.50%)	
Low levels of fruit and vegetable consumption, N (%)					<0.0001
No	2123 (61.38%)	1942 (61.20%)	2888 (54.24%)	6142 (61.64%)	
Yes	1336 (38.62%)	1231 (38.80%)	2436 (45.76%)	3823 (38.36%)	
High level of salt consumption, N (%)					0.0001
No	1769 (51.14%)	1551 (48.88%)	2846 (53.46%)	5013 (50.31%)	
Yes	1690 (48.86%)	1622 (51.12%)	2478 (46.54%)	4952 (49.69%)	

Characteristic	General deprivation**				p value[a]
	Q1 (N=3459)	Q2 (N=3173)	Q3 (N=5324)	Q4 (N=9965)	
High level of dairy fat consumption, N (%)					<0.0001
No	2892 (83.61%)	2638 (83.14%)	4537 (85.22%)	7535 (75.61%)	
Yes	567 (16.39%)	535 (16.86%)	787 (14.78%)	2430 (24.39%)	
Diabetes mellitus, N (%)					<0.0001
No	3205 (92.66%)	2816 (88.75%)	4882 (91.70%)	9132 (91.64%)	
Yes	254 (7.34%)	357 (11.25%)	442 (8.30%)	833 (8.36%)	
Prediabetes, N (%)					<0.0001
No	2912 (84.19%)	2519 (79.39%)	4367 (82.02%)	8473 (85.03%)	
Yes	547 (15.81%)	654 (20.61%)	957 (17.98%)	1492 (14.97%)	
Hypertension, N (%)					0.0100

Characteristic	General deprivation**				p value[a]
	Q1 (N=3459)	Q2 (N=3173)	Q3 (N=5324)	Q4 (N=9965)	
No	1761 (50.91%)	1579 (49.76%)	2663 (50.02%)	4786 (48.03%)	
Yes	1698 (49.09%)	1594 (50.24%)	2661 (49.98%)	5179 (51.97%)	
Elevated blood pressure, N (%)					<0.0001
No	3074 (88.87%)	3019 (95.15%)	4724 (88.73%)	9008 (90.40%)	
Yes	385 (11.13%)	154 (4.85%)	600 (11.27%)	957 (9.60%)	
Obesity, N (%)					<0.0001
No	2383 (68.89%)	2116 (66.69%)	3727 (70.00%)	6510 (65.33%)	
Yes	1076 (31.11%)	1057 (33.31%)	1597 (30.00%)	3455 (34.67%)	
Chronic kidney disease, N (%)					0.1550
No	3438 (99.39%)	3147 (99.18%)	5289 (99.34%)	9872 (99.07%)	
Yes	21 (0.61%)	26 (0.82%)	35 (0.66%)	93 (0.93%)	

Characteristic	General deprivation**				p value[a]
	Q1 (N=3459)	Q2 (N=3173)	Q3 (N=5324)	Q4 (N=9965)	
Hyperuricemia, N (%)					<0.0001
No	2816 (81.41%)	2705 (85.25%)	4202 (78.93%)	8317 (83.46%)	
Yes	643 (18.59%)	468 (14.75%)	1122 (21.07%)	1648 (16.54%)	
SBP (mmHg), Med [IQR]	131.0 [120.0;145.0]	130.0 [120.0;148.0]	133.0 [121.0;147.0]	130.0 [120.0;145.0]	<0.0001
DBP (mmHg), Med [IQR]	81.0 [75.0;90.0]	80.0 [72.0;89.0]	81.0 [73.0;90.0]	81.0 [75.0;90.0]	<0.0001
Fasting glucose (mmol/L), Med [IQR]	5.2 [4.8;5.6]	5.2 [4.6;5.8]	5.2 [4.8;5.6]	5.0 [4.4;5.5]	<0.0001
Creatinine (mg/dL), Med [IQR]	0.8 [0.7;0.9]	0.7 [0.7;0.8]	0.8 [0.7;0.8]	0.8 [0.7;0.8]	<0.0001
Serum uric acid (μmol/L), Med [IQR]	300.0 [247.0;369.0]	297.0 [242.2;352.0]	310.6 [252.6;380.0]	290.0 [230.0;350.0]	<0.0001

Characteristic	General deprivation**				p value[a]
	Q1 (N=3459)	Q2 (N=3173)	Q3 (N=5324)	Q4 (N=9965)	
Body Mass Index (kg/m^2), Med [IQR]	27.4 [23.9;31.1]	27.5 [24.3;31.5]	27.1 [23.7;30.9]	27.7 [24.1;31.8]	<0.0001
Antihypertensive medication, N (%)					<0.0001
No	2448 (70.77%)	2081 (65.58%)	3891 (73.08%)	6820 (68.44%)	
Yes	1011 (29.23%)	1092 (34.42%)	1433 (26.92%)	3145 (31.56%)	
Antidiabetic medication, N (%)					0.0003
No	3327 (96.18%)	3000 (94.55%)	5066 (95.15%)	9572 (96.06%)	
Yes	132 (3.82%)	173 (5.45%)	258 (4.85%)	393 (3.94%)	

Med: median; Q: quartile; IQR: interquartile range; SBP: systolic blood pressure; DBP: diastolic blood pressure. *Q1—the least deprived areas; Q4—the most deprived areas. **The Russian deprivation index measures general deprivation, and its components measure social, economic and environmental deprivation, respectively. [a]Categorical variables comparison by Pearson's Chi-squared test and continuous variables comparison by Kruskal-Wallis rank sum test. Significance set at a 2-sided $p < .05$.

Table S7: Baseline characteristics of study participants by quartile* of social deprivation.

Characteristic	Social deprivation**				p value[a]
	Q1 (N=5044)	Q2 (N=3695)	Q3 (N=7787)	Q4 (N=5395)	
Place of residence, N (%)					<0.0001
Urban	4519 (89.59%)	2725 (73.75%)	6224 (79.93%)	4260 (78.96%)	
Rural	525 (10.41%)	970 (26.25%)	1563 (20.07%)	1135 (21.04%)	
Sex, N (%)					<0.0001
Men	1833 (36.34%)	1562 (42.27%)	3132 (40.22%)	1848 (34.25%)	
Women	3211 (63.66%)	2133 (57.73%)	4655 (59.78%)	3547 (65.75%)	
Age (years), Med [IQR]	50.0 [39.0;57.0]	46.0 [35.0;55.0]	47.0 [35.0;56.0]	50.0 [39.0;57.0]	<0.0001
Marital status, N (%)					0.0001
Single	729 (14.45%)	558 (15.10%)	1246 (16.00%)	771 (14.29%)	
Married	3217 (63.78%)	2407 (65.14%)	5051 (64.86%)	3484 (64.58%)	
Windowed	694 (13.76%)	504 (13.64%)	980 (12.59%)	698 (12.94%)	
Divorced	404 (8.01%)	226 (6.12%)	510 (6.55%)	442 (8.19%)	
Income, N (%)					<0.0001
Low income	560 (11.10%)	379 (10.26%)	1036 (13.30%)	1031 (19.11%)	
Average income	3619 (71.75%)	2790 (75.51%)	5668 (72.79%)	3864 (71.62%)	

Characteristic	Social deprivation**				p value[a]
	Q1 (N=5044)	Q2 (N=3695)	Q3 (N=7787)	Q4 (N=5395)	
Higher income	865 (17.15%)	526 (14.24%)	1083 (13.91%)	500 (9.27%)	
Achieved education, N (%)					<0.0001
Primary level	695 (13.78%)	448 (12.12%)	1343 (17.25%)	1172 (21.72%)	
Secondary level	1891 (37.49%)	1317 (35.64%)	2799 (35.94%)	1905 (35.31%)	
Higher level	2458 (48.73%)	1930 (52.23%)	3645 (46.81%)	2318 (42.97%)	
Smoking status, N (%)					<0.0001
Never	3031 (60.09%)	2199 (59.51%)	4453 (57.19%)	3634 (67.36%)	
Former	922 (18.28%)	736 (19.92%)	1432 (18.39%)	687 (12.73%)	
Current	1091 (21.63%)	760 (20.57%)	1902 (24.43%)	1074 (19.91%)	
Alcohol drinking status, N (%)					<0.0001
Never	915 (18.14%)	931 (25.20%)	1637 (21.02%)	1863 (34.53%)	
Moderate	3944 (78.19%)	2646 (71.61%)	5930 (76.15%)	3350 (62.09%)	
Hard–drinking	185 (3.67%)	118 (3.19%)	220 (2.83%)	182 (3.37%)	
High level of sugar consumption, N (%)					<0.0001
No	2483 (49.23%)	2008 (54.34%)	3935 (50.53%)	2854 (52.90%)	

Characteristic	Social deprivation[**]				p value[a]
	Q1 (N=5044)	Q2 (N=3695)	Q3 (N=7787)	Q4 (N=5395)	
Yes	2561 (50.77%)	1687 (45.66%)	3852 (49.47%)	2541 (47.10%)	
Low levels of fruit and vegetable consumption, N (%)					<0.0001
No	3238 (64.20%)	2105 (56.97%)	4350 (55.86%)	3402 (63.06%)	
Yes	1806 (35.80%)	1590 (43.03%)	3437 (44.14%)	1993 (36.94%)	
High level of salt consumption, N (%)					0.0228
No	2627 (52.08%)	1912 (51.75%)	3864 (49.62%)	2776 (51.46%)	
Yes	2417 (47.92%)	1783 (48.25%)	3923 (50.38%)	2619 (48.54%)	
High level of dairy fat consumption, N (%)					<0.0001
No	4235 (83.96%)	3120 (84.44%)	5928 (76.13%)	4319 (80.06%)	
Yes	809 (16.04%)	575 (15.56%)	1859 (23.87%)	1076 (19.94%)	
Diabetes mellitus, N (%)					0.0024
No	4544 (90.09%)	3392 (91.80%)	7142 (91.72%)	4957 (91.88%)	
Yes	500 (9.91%)	303 (8.20%)	645 (8.28%)	438 (8.12%)	
Prediabetes, N (%)					<0.0001

Characteristic	Social deprivation[**]				p value[a]
	Q1 (N=5044)	Q2 (N=3695)	Q3 (N=7787)	Q4 (N=5395)	
No	3938 (78.07%)	3263 (88.31%)	6268 (80.49%)	4802 (89.01%)	
Yes	1106 (21.93%)	432 (11.69%)	1519 (19.51%)	593 (10.99%)	
Hypertension, N (%)					<0.0001
No	2254 (44.69%)	2114 (57.21%)	3973 (51.02%)	2448 (45.38%)	
Yes	2790 (55.31%)	1581 (42.79%)	3814 (48.98%)	2947 (54.62%)	
Elevated blood pressure, N (%)					<0.0001
No	4600 (91.20%)	3398 (91.96%)	6966 (89.46%)	4861 (90.10%)	
Yes	444 (8.80%)	297 (8.04%)	821 (10.54%)	534 (9.90%)	
Obesity, N (%)					<0.0001
No	3266 (64.75%)	2689 (72.77%)	5445 (69.92%)	3336 (61.84%)	
Yes	1778 (35.25%)	1006 (27.23%)	2342 (30.08%)	2059 (38.16%)	
Chronic kidney disease, N (%)					0.2160
No	5003 (99.19%)	3671 (99.35%)	7731 (99.28%)	5341 (99.00%)	
Yes	41 (0.81%)	24 (0.65%)	56 (0.72%)	54 (1.00%)	
Hyperuricemia					<0.0001

Characteristic	Social deprivation**				p value[a]
	Q1 (N=5044)	Q2 (N=3695)	Q3 (N=7787)	Q4 (N=5395)	
No	4103 (81.34%)	2985 (80.78%)	6316 (81.11%)	4636 (85.93%)	
Yes	941 (18.66%)	710 (19.22%)	1471 (18.89%)	759 (14.07%)	
SBP (mmHg), Med [IQR]	133.0 [120.0;150.0]	129.0 [118.0;142.0]	130.0 [119.0;144.0]	131.0 [120.0;145.0]	<0.0001
DBP (mmHg), Med [IQR]	81.0 [75.0;90.0]	78.0 [72.0;85.0]	81.0 [74.0;90.0]	82.0 [75.0;90.0]	<0.0001
Fasting glucose (mmol/L), Med [IQR]	5.3 [4.9;5.8]	5.0 [4.6;5.5]	5.2 [4.8;5.7]	4.8 [4.3;5.4]	<0.0001
Creatinine (mg/dL), Med [IQR]	0.8 [0.7;0.9]	0.7 [0.7;0.8]	0.7 [0.7;0.8]	0.8 [0.7;0.9]	<0.0001
Serum uric acid (μmol/L), Med [IQR]	305.5 [250.0;367.0]	300.0 [240.0;370.0]	300.0 [242.4;370.0]	280.0 [226.6;340.0]	<0.0001
Body Mass Index (kg/m^2), Med [IQR]	27.9 [24.2;31.8]	26.7 [23.7;30.4]	27.0 [23.6;31.0]	28.1 [24.5;32.4]	<0.0001
Antihypertensive medication, N (%)					<0.0001
No	3380 (67.01%)	2701 (73.10%)	5555 (71.34%)	3604 (66.80%)	
Yes	1664 (32.99%)	994 (26.90%)	2232 (28.66%)	1791 (33.20%)	
Antidiabetic medication, N (%)					0.0257

Characteristic	Social deprivation**				p value[a]
	Q1 (N=5044)	Q2 (N=3695)	Q3 (N=7787)	Q4 (N=5395)	
No	4816 (95.48%)	3506 (94.88%)	7454 (95.72%)	5189 (96.18%)	
Yes	228 (4.52%)	189 (5.12%)	333 (4.28%)	206 (3.82%)	

Med: median; Q: quartile; IQR: interquartile range; SBP: systolic blood pressure; DBP: diastolic blood pressure. *Q1—the least deprived areas; Q4—the most deprived areas. **The Russian deprivation index measures general deprivation, and its components measure social, economic and environmental deprivation, respectively. [a]Categorical variables comparison by Pearson's Chi-squared test and continuous variables comparison by Kruskal-Wallis rank sum test. Significance set at a 2-sided p < .05.

Table S8: Baseline characteristics of study participants by quartile* of economic deprivation.

Characteristic	Economic deprivation**				p value[a]
	Q1 (N=5321)	Q2 (N=6298)	Q3 (N=8717)	Q4 (N=1585)	
Place of residence, N (%)					<0.0001
Urban	4698 (88.29%)	5051 (80.20%)	6594 (75.65%)	1385 (87.38%)	
Rural	623 (11.71%)	1247 (19.80%)	2123 (24.35%)	200 (12.62%)	
Sex, N(%)					<0.0001
Men	1929 (36.25%)	2305 (36.60%)	3561 (40.85%)	580 (36.59%)	
Women	3392 (63.75%)	3993 (63.40%)	5156 (59.15%)	1005 (63.41%)	

Characteristic	Economic deprivation**				p value[a]
	Q1 (N=5321)	Q2 (N=6298)	Q3 (N=8717)	Q4 (N=1585)	
Age (years), Med [IQR]	48.0 [37.0;56.0]	49.0 [37.0;57.0]	47.0 [36.0;56.0]	53.0 [42.0;59.0]	<0.0001
Marital status, N (%)					<0.0001
Single	876 (16.46%)	928 (14.73%)	1264 (14.50%)	236 (14.89%)	
Married	3448 (64.80%)	4036 (64.08%)	5715 (65.56%)	960 (60.57%)	
Windowed	640 (12.03%)	852 (13.53%)	1186 (13.61%)	198 (12.49%)	
Divorced	357 (6.71%)	482 (7.65%)	552 (6.33%)	191 (12.05%)	
Income, N (%)					<0.0001
Low income	1044 (19.62%)	813 (12.91%)	942 (10.81%)	207 (13.06%)	
Average income	3633 (68.28%)	4945 (78.52%)	6125 (70.26%)	1238 (78.11%)	
Higher income	644 (12.10%)	540 (8.57%)	1650 (18.93%)	140 (8.83%)	
Achieved education, N (%)					<0.0001
Primary level	852 (16.01%)	1277 (20.28%)	1243 (14.26%)	286 (18.04%)	
Secondary level	1627 (30.58%)	2521 (40.03%)	3100 (35.56%)	664 (41.89%)	
Higher level	2842 (53.41%)	2500 (39.70%)	4374 (50.18%)	635 (40.06%)	
Smoking status, N (%)					<0.0001

Characteristic	Economic deprivation**				p value[a]
	Q1 (N=5321)	Q2 (N=6298)	Q3 (N=8717)	Q4 (N=1585)	
Never	3480 (65.40%)	4020 (63.83%)	4772 (54.74%)	1045 (65.93%)	
Former	802 (15.07%)	881 (13.99%)	1906 (21.87%)	188 (11.86%)	
Current	1039 (19.53%)	1397 (22.18%)	2039 (23.39%)	352 (22.21%)	
Alcohol drinking status, N (%)					<0.0001
Never	1822 (34.24%)	1472 (23.37%)	1599 (18.34%)	453 (28.58%)	
Moderate	3339 (62.75%)	4621 (73.37%)	6788 (77.87%)	1122 (70.79%)	
Hard–drinking	160 (3.01%)	205 (3.26%)	330 (3.79%)	10 (0.63%)	
High level of sugar consumption, N (%)					<0.0001
No	2917 (54.82%)	3238 (51.41%)	4281 (49.11%)	844 (53.25%)	
Yes	2404 (45.18%)	3060 (48.59%)	4436 (50.89%)	741 (46.75%)	
Low levels of fruit and vegetable consumption, N (%)					<0.0001
No	3117 (58.58%)	3972 (63.07%)	4891 (56.11%)	1115 (70.35%)	
Yes	2204 (41.42%)	2326 (36.93%)	3826 (43.89%)	470 (29.65%)	
High level of salt consumption, N (%)					<0.0001

Characteristic	Economic deprivation**				p value[a]
	Q1 (N=5321)	Q2 (N=6298)	Q3 (N=8717)	Q4 (N=1585)	
No	2562 (48.15%)	3197 (50.76%)	4562 (52.33%)	858 (54.13%)	
Yes	2759 (51.85%)	3101 (49.24%)	4155 (47.67%)	727 (45.87%)	
High level of dairy fat consumption, N (%)					<0.0001
No	4513 (84.81%)	4599 (73.02%)	7147 (81.99%)	1343 (84.73%)	
Yes	808 (15.19%)	1699 (26.98%)	1570 (18.01%)	242 (15.27%)	
Diabetes mellitus, N (%)					<0.0001
No	4890 (91.90%)	5784 (91.84%)	8022 (92.03%)	1339 (84.48%)	
Yes	431 (8.10%)	514 (8.16%)	695 (7.97%)	246 (15.52%)	
Prediabetes, N (%)					<0.0001
No	4875 (91.62%)	5339 (84.77%)	7031 (80.66%)	1026 (64.73%)	
Yes	446 (8.38%)	959 (15.23%)	1686 (19.34%)	559 (35.27%)	
Hypertension, N (%)					<0.0001
No	3057 (57.45%)	2967 (47.11%)	4272 (49.01%)	493 (31.10%)	
Yes	2264 (42.55%)	3331 (52.89%)	4445 (50.99%)	1092 (68.90%)	
Elevated blood pressure, N (%)					<0.0001

Characteristic	Economic deprivation**				p value[a]
	Q1 (N=5321)	Q2 (N=6298)	Q3 (N=8717)	Q4 (N=1585)	
No	4944 (92.91%)	5680 (90.19%)	7675 (88.05%)	1526 (96.28%)	
Yes	377 (7.09%)	618 (9.81%)	1042 (11.95%)	59 (3.72%)	
Obesity, N (%)					<0.0001
No	3707 (69.67%)	4178 (66.34%)	5968 (68.46%)	883 (55.71%)	
Yes	1614 (30.33%)	2120 (33.66%)	2749 (31.54%)	702 (44.29%)	
Chronic kidney disease, N (%)					0.0002
No	5294 (99.49%)	6228 (98.89%)	8659 (99.33%)	1565 (98.74%)	
Yes	27 (0.51%)	70 (1.11%)	58 (0.67%)	20 (1.26%)	
Hyperuricemia, N (%)					<0.0001
No	4649 (87.37%)	5344 (84.85%)	6760 (77.55%)	1287 (81.20%)	
Yes	672 (12.63%)	954 (15.15%)	1957 (22.45%)	298 (18.80%)	
SBP (mmHg), Med [IQR]	125.0 [116.0;140.0]	130.0 [120.0;145.0]	133.0 [121.0;147.0]	140.0 [127.0;160.0]	<0.0001
DBP (mmHg), Med [IQR]	79.0 [72.0;85.0]	82.0 [75.0;90.0]	82.0 [74.0;90.0]	84.0 [77.0;90.0]	<0.0001
Fasting glucose (mmol/L), Med [IQR]	4.7 [4.3;5.3]	5.0 [4.5;5.5]	5.2 [4.9;5.7]	5.6 [5.2;6.3]	<0.0001

Characteristic	Economic deprivation**				p value[a]
	Q1 (N=5321)	Q2 (N=6298)	Q3 (N=8717)	Q4 (N=1585)	
Creatinine (mg/dL), Med [IQR]	0.7 [0.7;0.8]	0.8 [0.7;0.9]	0.8 [0.7;0.9]	0.8 [0.7;0.9]	<0.0001
Serum uric acid (μmol/L), Med [IQR]	279.0 [221.0;340.0]	290.0 [230.0;350.0]	310.0 [250.7;380.0]	311.3 [263.9;366.1]	<0.0001
Body Mass Index (kg/m^2), Med [IQR]	27.1 [23.9;31.0]	27.6 [24.1;31.6]	27.3 [23.8;31.2]	29.1 [25.4;33.3]	<0.0001
Antihypertensive medication, N (%)					<0.0001
No	3728 (70.06%)	4345 (68.99%)	6235 (71.53%)	932 (58.80%)	
Yes	1593 (29.94%)	1953 (31.01%)	2482 (28.47%)	653 (41.20%)	
Antidiabetic medication, N (%)					0.0019
No	5083 (95.53%)	6055 (96.14%)	8338 (95.65%)	1489 (93.94%)	
Yes	238 (4.47%)	243 (3.86%)	379 (4.35%)	96 (6.06%)	

Med: median; Q: quartile; IQR: interquartile range; SBP: systolic blood pressure; DBP: diastolic blood pressure. *Q1—the least deprived areas; Q4—the most deprived areas. **The Russian deprivation index measures general deprivation, and its components measure social, economic and environmental deprivation, respectively. [a]Categorical variables comparison by Pearson's Chi-squared test and continuous variables comparison by Kruskal-Wallis rank sum test. Significance set at a 2-sided $p < .05$.

Table S9: Baseline characteristics of study participants by quartile* of environmental deprivation.

Characteristic	Environmental deprivation**				p value[a]
	Q1 (N=3733)	Q2 (N=1872)	Q3 (N=6244)	Q4 (N=10072)	
Place of residence, N (%)					<0.0001
Urban	3307 (88.59%)	1547 (82.64%)	5067 (81.15%)	7807 (77.51%)	
Rural	426 (11.41%)	325 (17.36%)	1177 (18.85%)	2265 (22.49%)	
Sex, N (%)					<0.0001
Men	1231 (32.98%)	686 (36.65%)	2405 (38.52%)	4053 (40.24%)	
Women	2502 (67.02%)	1186 (63.35%)	3839 (61.48%)	6019 (59.76%)	
Age (years), Med [IQR]	49.0 [39.0;57.0]	48.0 [37.0;56.0]	49.0 [37.0;57.0]	48.0 [36.0;56.0]	<0.0001
Marital status, N (%)					<0.0001
Single	627 (16.80%)	212 (11.32%)	937 (15.01%)	1528 (15.17%)	
Married	2396 (64.18%)	1240 (66.24%)	4014 (64.29%)	6509 (64.62%)	
Windowed	449 (12.03%)	285 (15.22%)	809 (12.96%)	1333 (13.23%)	
Divorced	261 (6.99%)	135 (7.21%)	484 (7.75%)	702 (6.97%)	
Income, N (%)					<0.0001
Low income	802 (21.48%)	187 (9.99%)	973 (15.58%)	1044 (10.37%)	
Average income	2425 (64.96%)	1330 (71.05%)	4592 (73.54%)	7594 (75.40%)	

Characteristic	Environmental deprivation**				p value[a]
	Q1 (N=3733)	Q2 (N=1872)	Q3 (N=6244)	Q4 (N=10072)	
Higher income	506 (13.55%)	355 (18.96%)	679 (10.87%)	1434 (14.24%)	
Achieved education, N (%)					<0.0001
Primary level	641 (17.17%)	269 (14.37%)	1135 (18.18%)	1613 (16.01%)	
Secondary level	1011 (27.08%)	776 (41.45%)	2400 (38.44%)	3725 (36.98%)	
Higher level	2081 (55.75%)	827 (44.18%)	2709 (43.39%)	4734 (47.00%)	
Smoking status, N (%)					<0.0001
Never	2428 (65.04%)	1186 (63.35%)	3867 (61.93%)	5836 (57.94%)	
Former	567 (15.19%)	340 (18.16%)	969 (15.52%)	1901 (18.87%)	
Current	738 (19.77%)	346 (18.48%)	1408 (22.55%)	2335 (23.18%)	
Alcohol drinking status, N (%)					<0.0001
Never	1517 (40.64%)	274 (14.64%)	1431 (22.92%)	2124 (21.09%)	
Moderate	2117 (56.71%)	1485 (79.33%)	4606 (73.77%)	7662 (76.07%)	
Hard–drinking	99 (2.65%)	113 (6.04%)	207 (3.32%)	286 (2.84%)	
High level of sugar consumption, N (%)					<0.0001
No	2092 (56.04%)	836 (44.66%)	3415 (54.69%)	4937 (49.02%)	

Characteristic	Environmental deprivation**				p value[a]
	Q1 (N=3733)	Q2 (N=1872)	Q3 (N=6244)	Q4 (N=10072)	
Yes	1641 (43.96%)	1036 (55.34%)	2829 (45.31%)	5135 (50.98%)	
Low levels of fruit and vegetable consumption, N (%)					<0.0001
No	2290 (61.34%)	1075 (57.43%)	3344 (53.56%)	6386 (63.40%)	
Yes	1443 (38.66%)	797 (42.57%)	2900 (46.44%)	3686 (36.60%)	
High level of salt consumption, N (%)					<0.0001
No	1869 (50.07%)	923 (49.31%)	2924 (46.83%)	5463 (54.24%)	
Yes	1864 (49.93%)	949 (50.69%)	3320 (53.17%)	4609 (45.76%)	
High level of dairy fat consumption, N (%)					<0.0001
No	3218 (86.20%)	1493 (79.75%)	5033 (80.61%)	7858 (78.02%)	
Yes	515 (13.80%)	379 (20.25%)	1211 (19.39%)	2214 (21.98%)	
Diabetes mellitus, N (%)					<0.0001
No	3413 (91.43%)	1734 (92.63%)	5547 (88.84%)	9341 (92.74%)	
Yes	320 (8.57%)	138 (7.37%)	697 (11.16%)	731 (7.26%)	
Prediabetes, N (%)					<0.0001

Characteristic	Environmental deprivation**				p value[a]
	Q1 (N=3733)	Q2 (N=1872)	Q3 (N=6244)	Q4 (N=10072)	
No	3382 (90.60%)	1528 (81.62%)	5096 (81.61%)	8265 (82.06%)	
Yes	351 (9.40%)	344 (18.38%)	1148 (18.39%)	1807 (17.94%)	
Hypertension, N (%)					<0.0001
No	1971 (52.80%)	875 (46.74%)	3106 (49.74%)	4837 (48.02%)	
Yes	1762 (47.20%)	997 (53.26%)	3138 (50.26%)	5235 (51.98%)	
Elevated blood pressure, N (%)					<0.0001
No	3451 (92.45%)	1592 (85.04%)	5807 (93.00%)	8975 (89.11%)	
Yes	282 (7.55%)	280 (14.96%)	437 (7.00%)	1097 (10.89%)	
Obesity, N (%)					0.0256
No	2474 (66.27%)	1224 (65.38%)	4279 (68.53%)	6759 (67.11%)	
Yes	1259 (33.73%)	648 (34.62%)	1965 (31.47%)	3313 (32.89%)	
Chronic kidney disease, N (%)					0.0257
No	3712 (99.44%)	1855 (99.09%)	6178 (98.94%)	10001 (99.30%)	
Yes	21 (0.56%)	17 (0.91%)	66 (1.06%)	71 (0.70%)	
Hyperuricemia, N (%)					<0.0001

Characteristic	Environmental deprivation**				p value[a]
	Q1 (N=3733)	Q2 (N=1872)	Q3 (N=6244)	Q4 (N=10072)	
No	3231 (86.55%)	1485 (79.33%)	5299 (84.87%)	8025 (79.68%)	
Yes	502 (13.45%)	387 (20.67%)	945 (15.13%)	2047 (20.32%)	
SBP (mmHg), Med [IQR]	127.0 [117.0;140.0]	133.0 [121.0;147.0]	130.0 [120.0;145.0]	133.0 [120.0;147.0]	<0.0001
DBP (mmHg), Med [IQR]	80.0 [72.0;87.0]	83.0 [78.0;92.0]	80.0 [73.0;90.0]	82.0 [75.0;90.0]	<0.0001
Fasting glucose (mmol/L), Med [IQR]	4.8 [4.3;5.3]	5.2 [4.9;5.6]	5.2 [4.6;5.8]	5.1 [4.7;5.6]	<0.0001
Creatinine (mg/dL), Med [IQR]	0.7 [0.7;0.8]	0.8 [0.8;0.9]	0.7 [0.7;0.8]	0.8 [0.7;0.8]	<0.0001
Serum uric acid (µmol/L), Med [IQR]	280.0 [220.0;344.0]	310.0 [250.0;370.0]	296.6 [242.1;353.4]	300.0 [240.0;370.0]	<0.0001
Body Mass Index (kg/m^2), Med [IQR]	27.5 [24.1;31.6]	27.9 [24.4;31.7]	27.2 [24.1;31.2]	27.4 [23.8;31.4]	0.0016
Antihypertensive medication, N (%)					<0.0001
No	2579 (69.09%)	1268 (67.74%)	4171 (66.80%)	7222 (71.70%)	
Yes	1154 (30.91%)	604 (32.26%)	2073 (33.20%)	2850 (28.30%)	
Antidiabetic medication, N (%)					<0.0001

Characteristic	Environmental deprivation**				p value[a]
	Q1 (N=3733)	Q2 (N=1872)	Q3 (N=6244)	Q4 (N=10072)	
No	3572 (95.69%)	1799 (96.10%)	5890 (94.33%)	9704 (96.35%)	
Yes	161 (4.31%)	73 (3.90%)	354 (5.67%)	368 (3.65%)	

Med: median; Q: quartile; IQR: interquartile range; SBP: systolic blood pressure; DBP: diastolic blood pressure. *Q1—the least deprived areas; Q4—the most deprived areas. **The Russian deprivation index measures general deprivation, and its components measure social, economic and environmental deprivation, respectively. [a] Categorical variables comparison by Pearson's Chi-squared test and continuous variables comparison by Kruskal-Wallis rank sum test. Significance set at a 2-sided $p < .05$.

Table 10: Association of general deprivation with baseline blood pressure (SBP and DBP), creatinine, fasting glucose, uric acid levels and body mass index.

		General deprivation		
Dependent variables	Level of deprivation	Total population β (95% CI)	Men β (95% CI)	Women β (95% CI)
SBP (mmHg)	Q1	REF		
	Q2	-0.77 (-10.56 to 9.02)	-2.32 (-12.74 to 8.10)	0.59 (-8.36 to 9.55)
	Q3	3.00 (-0.63 to 6.64)	**4.39 (2.70 to 6.07)**	2.16 (-2.54 to 6.86)
	Q4	-1.54 (-5.72 to 2.65)	0.26 (-2.02 to 2.54)	-2.43 (-7.67 to 2.81)
DBP (mmHg)	Q1	REF		
	Q2	-1.82 (-6.75 to 3.11)	-2.51 (-7.18 to 2.17)	-1.30 (-6.21 to 3.60)
	Q3	-0.24 (-3.24 to 2.75)	0.47 (-1.77 to 2.70)	-0.79 (-4.21 to 2.63)
	Q4	0.13 (-3.01 to 3.26)	0.62 (-1.92 to 3.17)	-0.19 (-3.70 to 3.31)
Fasting glucose (mmol/L)	Q1	REF		
	Q2	0.06 (-0.70 to 0.82)	0.02 (-0.72 to 0.76)	0.10 (-0.66 to 0.86)
	Q3	0.05 (-0.13 to 0.23)	0.07 (-0.06 to 0.21)	0.04 (-0.16 to 0.25)
	Q4	-0.18 (-0.45 to 0.09)	-0.15 (-0.38 to 0.08)	-0.19 (-0.49 to 0.10)
Serum uric acid (μmol/L)	Q1	REF		
	Q2	-10.56 (-35.72 to 14.59)	-26.01 (-53.51 to 1.48)	1.27 (-19.85 to 22.38)
	Q3	8.19 (-5.77 to 22.14)	7.22 (-12.87 to 27.31)	8.44 (-4.64 to 21.52)
	Q4	-9.69	-16.19	-4.05

		(-27.44 to 8.05)	(-33.00 to 0.62)	(-23.09 to 15.00)
Creatinine (mg/dL)	Q1	REF		
	Q2	-0.06 (-0.15 to 0.04)	-0.08 (-0.20 to 0.03)	-0.04 (-0.12 to 0.04)
	Q3	-0.03 (-0.09 to 0.03)	-0.03 (-0.11 to 0.04)	-0.03 (-0.09 to 0.02)
	Q4	-0.03 (-0.09 to 0.04)	-0.04 (-0.12 to 0.04)	-0.02 (-0.08 to 0.05)
Body Mass Index (kg/m^2)	Q1	REF		
	Q2	0.20 (-1.29 to 1.70)	0.08 (-1.29 to 1.70)	0.42 (-1.34 to 2.18)
	Q3	-0.16 (-0.94 to 0.61)	-0.17 (-0.94 to 0.61)	-0.08 (-0.99 to 0.83)
	Q4	0.19 (-0.68 to 1.05)	-0.04 (-0.68 to 1.05)	0.48 (-0.48 to 1.44)

Q: quartile; SBP: systolic blood pressure; DBP: diastolic blood pressure; REF: Reference category. Q1—the least deprived areas; Q4—the most deprived areas. Boldface indicates statistical significance ($p < 0.05$). All values were adjusted for age, sex, marital status, income and education, smoking and alcohol drinking status, place of residence, dietary habits (sugar, salt, milk fat, vegetables and fruits intake). Models for fasting glucose additionally included whether the individual was prescribed any antidiabetic medications, and models for blood pressure similarly included a variable for the use of antihypertensive medications.

Table S11: Association of social deprivation with baseline blood pressure (SBP and DBP), creatinine, fasting glucose, uric acid levels and body mass index.

		Social deprivation		
Dependent variables	Level of deprivation	Total population β (95% CI)	Men β (95% CI)	Women β (95% CI)
SBP (mmHg)	Q1	REF		
	Q2	-3.11 (-11.34 to 5.11)	-3.41 (-13.25 to 6.43)	-2.55 (-9.56 to 4.45)
	Q3	-2.31 (-7.11 to 2.48)	-0.15 (-4.37 to 4.06)	-3.43 (-8.65 to 1.78)

	Q4	-2.67 (-8.03 to 2.69)	-0.87 (-4.42 to 2.68)	-3.58 (-10.06 to 2.89)
DBP (mmHg)	Q1	REF		
	Q2	**-3.32 (-6.22 to -0.42)**	-3.14 (-6.51 to 0.22)	**-3.36 (-6.02 to -0.69)**
	Q3	-0.33 (-2.52 to 1.85)	0.35 (-1.33 to 2.04)	-0.74 (-3.21 to 1.72)
	Q4	0.08 (-2.92 to 3.07)	0.92 (-1.87 to 3.71)	-0.40 (-3.68 to 2.87)
Fasting glucose (mmol/L)	Q1	REF		
	Q2	-0.38 (-0.85 to 0.08)	-0.38 (-0.85 to 0.10)	-0.38 (-0.84 to 0.08)
	Q3	-0.16 (-0.51 to 0.18)	-0.12 (-0.45 to 0.22)	-0.19 (-0.54 to 0.17)
	Q4	**-0.54 (-0.90 to -0.17)**	**-0.54 (-0.87 to -0.21)**	**-0.53 (-0.93 to -0.13)**
Serum uric acid (µmol/L)	Q1	REF		
	Q2	-4.16 (-34.36 to 26.03)	-3.20 (-49.60 to 43.21)	-4.08 (-21.76 to 13.61)
	Q3	-1.40 (-16.87 to 14.07)	-4.54 (-19.04 to 9.97)	2.41 (-15.98 to 20.80)
	Q4	**-19.28 (-29.67 to -8.88)**	**-20.52 (-30.48 to -10.55)**	**-17.48 (-31.54 to -3.41)**
Creatinine (mg/dL)	Q1	REF		
	Q2	-0.06 (-0.13 to 0.009)	-0.07 (-0.16 to 0.02)	**-0.06 (-0.11 to -0.00004)**
	Q3	-0.04	-0.05	-0.04

		(-0.09 to 0.007)	(-0.11 to 0.003)	(-0.09 to 0.02)
	Q4	-0.001<br>(-0.05 to 0.04)	-0.003<br>(-0.05 to 0.05)	0.001<br>(-0.04 to 0.04)
Body Mass Index (kg/m^2)	Q1	REF		
	Q2	-0.78<br>(-1.62 to 0.06)	-0.33<br>(-0.86 to 0.19)	-0.99<br>(-1.98 to 0.004)
	Q3	-0.55<br>(-1.36 to 0.25)	**-0.51**<br>**(-1.01 to -0.01)**	-0.40<br>(-1.42 to 0.62)
	Q4	0.16<br>(-0.64 to 0.97)	0.005<br>(-0.61 to 0.62)	0.33<br>(-0.66 to 1.33)

Q: quartile; SBP: systolic blood pressure; DBP: diastolic blood pressure; REF: Reference category. Q1—the least deprived areas; Q4—the most deprived areas. Boldface indicates statistical significance ($p < 0.05$). All values were adjusted for age, sex, marital status, income and education, smoking and alcohol drinking status, place of residence, dietary habits (sugar, salt, milk fat, vegetables and fruits intake). Models for fasting glucose additionally included whether the individual was prescribed any antidiabetic medications, and models for blood pressure similarly included a variable for the use of antihypertensive medications.

Table S12: Association of economic deprivation with baseline blood pressure (SBP and DBP), creatinine, fasting glucose, uric acid levels and body mass index.

		Economic deprivation		
Dependent variables	Level of deprivation	Total population<br>β (95% CI)	Men<br>β (95% CI)	Women<br>β (95% CI)
SBP (mmHg)	Q1	REF		
	Q2	3.87<br>(-0.15 to 7.88)	3.86<br>(-1.26 to 8.98)	3.72<br>(-0.62 to 8.05)
	Q3	**7.51**<br>**(4.95 to 10.07)**	**7.50**<br>**(2.70 to 12.29)**	**7.42**<br>**(5.21 to 9.62)**
	Q4	**11.02**<br>**(8.87 to 13.17)**	**10.02**<br>**(5.48 to 14.57)**	**11.44**<br>**(9.79 to 13.09)**
DBP (mmHg)	Q1	REF		
	Q2	**3.49**	**3.58**	**3.41**

		**(1.21 to 5.77)**	**(0.40 to 6.75)**	**(1.49 to 5.32)**
	Q3	**3.42 (1.69 to 5.16)**	**3.45 (1.00 to 5.89)**	**3.38 (1.88 to 4.89)**
	Q4	**4.07 (2.70 to 5.45)**	**3.57 (1.20 to 5.95)**	**4.31 (3.52 to 5.10)**
Fasting glucose (mmol/L)	Q1	REF		
	Q2	**0.23 (0.03 to 0.42)**	0.14 (-0.07 to 0.36)	**0.28 (0.07 to 0.48)**
	Q3	**0.51 (0.30 to 0.72)**	**0.45 (0.25 to 0.64)**	**0.55 (0.33 to 0.77)**
	Q4	**1.00 (0.81 to 1.18)**	**0.94 (0.77 to 1.11)**	**1.03 (0.84 to 1.23)**
Serum uric acid (μmol/L)	Q1	REF		
	Q2	7.50 (-1.99 to 17.00)	2.04 (-16.89 to 20.98)	**10.06 (1.34 to 18.79)**
	Q3	**32.67 (19.93 to 45.40)**	**33.21 (12.47 to 53.95)**	**31.17 (16.67 to 45.67)**
	Q4	**26.81 (17.91 to 35.71)**	**19.52 (2.87 to 36.16)**	**30.12 (22.05 to 38.19)**
Creatinine (mg/dL)	Q1	REF		
	Q2	0.04 (-0.02 to 0.10)	0.05 (-0.02 to 0.11)	0.04 (-0.02 to 0.09)
	Q3	0.03 (-0.02 to 0.09)	0.05 (-0.01 to 0.12)	0.02 (-0.02 to 0.07)
	Q4	**0.05 (0.01 to 0.09)**	**0.06 (0.01 to 0.11)**	**0.04 (0.01 to 0.08)**
Body Mass Index (kg/m^2)	Q1	REF		
	Q2	0.12	-0.16	0.25

		(-0.79 to 1.03)	(-0.80 to 0.48)	(-0.85 to 1.34)
	Q3	0.18	-0.02	0.26
		(-0.59 to 0.95)	(-0.59 to 0.55)	(-0.73 to 1.24)
	Q4	**1.26**	**0.67**	**1.49**
		**(0.50 to 2.01)**	**(0.15 to 1.18)**	**(0.55 to 2.43)**

Q: quartile; SBP: systolic blood pressure; DBP: diastolic blood pressure; REF: Reference category. Q1—the least deprived areas; Q4—the most deprived areas. Boldface indicates statistical significance ($p < 0.05$). All values were adjusted for age, sex, marital status, income and education, smoking and alcohol drinking status, place of residence, dietary habits (sugar, salt, milk fat, vegetables and fruits intake). Models for fasting glucose additionally included whether the individual was prescribed any antidiabetic medications, and models for blood pressure similarly included a variable for the use of antihypertensive medications.

Table S13: Association of environmental deprivation with baseline blood pressure (SBP and DBP), creatinine, fasting glucose, uric acid levels and body mass index.

		Environmental deprivation		
Dependent variables	Level of deprivation	Total population β (95% CI)	Men β (95% CI)	Women β (95% CI)
SBP (mmHg)	Q1	REF		
	Q2	**6.00**	**1.57**	**8.10**
		**(4.27 to 7.73)**	**(1.12 to 2.02)**	**(5.87 to 10.34)**
	Q3	2.29	-0.44	3.74
		(-3.17 to 7.74)	(-6.52 to 5.64)	(-1.62 to 9.09)
	Q4	**5.70**	**3.87**	**6.53**
		**(3.06 to 8.34)**	**(2.18 to 5.56)**	**(3.27 to 9.79)**
DBP (mmHg)	Q1	REF		
	Q2	**4.00**	**2.41**	**4.85**
		**(3.81 to 4.20)**	**(2.07 to 2.75)**	**(4.55 to 5.15)**
	Q3	0.88	-0.24	1.50
		(-1.46 to 3.22)	(-3.14 to 2.66)	(-0.54 to 3.53)
	Q4	**2.73**	**2.14**	**2.99**
		**(1.54 to 3.92)**	**(0.83 to 3.44)**	**(1.71 to 4.26)**

Fasting glucose (mmol/L)	Q1	REF		
	Q2	**0.49 (0.23 to 0.76)**	**0.35 (0.15 to 0.55)**	**0.56 (0.28 to 0.84)**
	Q3	0.39 (-0.07 to 0.85)	0.27 (-0.16 to 0.70)	0.45 (-0.01 to 0.92)
	Q4	**0.34 (0.05 to 0.62)**	0.24 (-0.01 to 0.49)	**0.38 (0.08 to 0.68)**
Serum uric acid (μmol/L)	Q1	REF		
	Q2	**25.83 (14.85 to 36.80)**	**20.62 (13.45 to 27.80)**	**26.14 (15.54 to 36.74)**
	Q3	9.80 (-7.65 to 27.26)	-8.75 (-25.68 to 8.18)	**20.25 (4.66 to 35.84)**
	Q4	**20.22 (3.94 to 36.51)**	12.90 (-4.82 to 30.62)	**23.13 (6.76 to 39.49)**
Creatinine (mg/dL)	Q1	REF		
	Q2	**0.07 (0.06 to 0.09)**	**0.09 (0.07 to 0.12)**	**0.06 (0.05 to 0.08)**
	Q3	-0.004 (-0.05 to 0.04)	-0.02 (-0.08 to 0.04)	0.006 (-0.04 to 0.05)
	Q4	-0.0001 (-0.03 to 0.03)	0.003 (-0.03 to 0.04)	-0.002 (-0.04 to 0.03)
Body Mass Index (kg/m^2)	Q1	REF		
	Q2	0.39 (-0.46 to 1.23)	0.19 (-0.53 to 0.91)	0.34 (-0.70 to 1.39)
	Q3	-0.25 (-1.41 to 0.91)	-0.36 (-1.23 to 0.51)	-0.18 (-1.55 to 1.19)
	Q4	-0.05 (-0.92 to 0.83)	-0.23 (-0.96 to 0.50)	0.06 (-1.03 to 1.15)

Q: quartile; SBP: systolic blood pressure; DBP: diastolic blood pressure; REF: Reference category. Q1—the least deprived areas; Q4—the most deprived areas. Boldface indicates statistical significance ($p < 0.05$). All values were adjusted for age, sex, marital status, income and education, smoking and alcohol drinking status, place of residence, dietary habits (sugar, salt, milk fat, vegetables and fruits intake). Models for fasting glucose additionally included whether the individual was prescribed any antidiabetic medications, and models for blood pressure similarly included a variable for the use of antihypertensive medications.

R code

The tables show a code for general deprivation. Identical code was written for social, economic and environmental deprivation (not shown).

AG: Hypertension, PREAG: Elevated blood pressure, SD: Diabetes mellitus, PRESD: Prediabetes, GFR: Chronic kidney disease, UR: Hyperuricemia, OB: Obesity, SBP: Systolic blood pressure, DBP: Diastolic blood pressure, UACIDE: Serum uric acid, CRE: Creatinine, GLU: Fasting glucose, IBM: Body Mass Index, INDEX: Deprivation index, SEX: Sex, AGE: Age, MARST: Marital status, INCOME: Income, EDUS: Achieved education, SMST: Smoking status, DRST: Alcohol drinking status, HSUG: High level of sugar consumption, LFVI: Low levels of fruit and vegetable consumption, HSALT: High level of salt consumption, HFAT: High level of dairy fat consumption, SETLR: Place of residence, DMDR2W: Taking antidiabetic medications, AHTRT: Taking antihypertensive medications, id: id federal subjecs.

Table S14: R code for total population.
Outcomes: Categorical variables – AG, PREAG, SD, PRESD, GFR, UR, OB.

Total population	
Deprivation index	
Model0	gee (AG ~ INDEX , data = df, family = binomial (link = "logit"), id = id, corstr = "independence", scale.fix = TRUE, scale.value = 1)<br><br>gee (PREAG ~ INDEX , data = df, family = binomial (link = "logit"), id = id, corstr = "independence", scale.fix = TRUE, scale.value = 1)<br><br>gee (PRESD ~ INDEX , data = df, family = binomial (link = "logit"), id = id, corstr = "independence", scale.fix = TRUE, scale.value = 1)<br><br>gee (SD ~ INDEX , data = df, family = binomial (link = "logit"), id = id, corstr = "independence", scale.fix = TRUE, scale.value = 1)<br><br>gee (GFR ~ INDEX , data = df, family = binomial (link = "logit"), id = id, corstr = "independence", scale.fix = TRUE, scale.value = 1)<br><br>gee (UR ~ INDEX , data = df, family = binomial (link = "logit"), id = id, corstr = "independence", scale.fix = TRUE, scale.value = 1)

	gee (OB~ INDEX , data = df, family = binomial (link = "logit"), id = id, corstr = "independence", scale.fix = TRUE, scale.value = 1)
Model1	gee (AG ~ INDEX + SETLR + HSUG + DRST + SMST + EDUS + MARST + SEX + AGE, data = df, family = binomial (link = "logit"), id = id, corstr = "independence", scale.fix = TRUE, scale.value = 1)<br><br>gee (PREAG ~ INDEX + SETLR + DRST + SMST + EDUS + INCOME + MARST + SEX + AGE, data = df, family = binomial (link = "logit"), id = id, corstr = "independence", scale.fix = TRUE, scale.value = 1)<br><br>gee (PRESD ~ INDEX + LFVI + HSUG + DRST + SMST + INCOME + MARST + SEX + AGE, data = df, family = binomial (link = "logit"), id = id, corstr = "independence", scale.fix = TRUE, scale.value = 1)<br><br>gee (SD ~ INDEX + LFVI + HSUG + DRST + SMST + INCOME + MARST + SEX + AGE, data = df, family = binomial (link = "logit"), id = id, corstr = "independence", scale.fix = TRUE, scale.value = 1)<br><br>gee (GFR ~ INDEX + SETLR + LFVI + HSUG + DRST + SMST + EDUS + INCOME + MARST, data = df, family = binomial (link = "logit"), id = id, corstr = "independence", scale.fix = TRUE, scale.value = 1)<br><br>gee (UR ~ INDEX + SETLR + HSALT + LFVI + HSUG + DRST + SMST + EDUS + INCOME + AGE, data = df, family = binomial (link = "logit"), id = id, corstr = "independence", scale.fix = TRUE, scale.value = 1)<br><br>gee (OB ~INDEX + SETLR + LFVI + HSUG + DRST + SMST + EDUS + INCOME + MARST + SEX + AGE, data = df, family = binomial (link = "logit"), id = id, corstr = "independence", scale.fix = TRUE, scale.value = 1)
Model2	gee (AG ~ INDEX + SEX + AGE + MARST+ INCOME + EDUS + SMST + DRST + HSUG + LFVI + HSALT + HFAT + SETLR, data = df, family = binomial (link = "logit"), id = id, corstr = "independence", scale.fix = TRUE, scale.value = 1)<br><br>gee (PREAG ~ INDEX + SEX + AGE + MARST+ INCOME + EDUS + SMST + DRST + HSUG + LFVI + HSALT + HFAT + SETLR, data = df, family = binomial (link = "logit"), id = id, corstr = "independence", scale.fix = TRUE, scale.value = 1)<br><br>gee (PRESD ~ INDEX + SEX + AGE + MARST+ INCOME + EDUS + SMST + DRST + HSUG + LFVI + HSALT + HFAT + SETLR, data = df, family = binomial (link = "logit"), id = id, corstr = "independence", scale.fix = TRUE, scale.value = 1)<br><br>gee (SD ~ INDEX + SEX + AGE + MARST+ INCOME + EDUS + SMST + DRST + HSUG + LFVI + HSALT + HFAT + SETLR, data = df, family = binomial (link = "logit"), id = id, corstr = "independence", scale.fix = TRUE, scale.value = 1)<br><br>gee (GFR ~ INDEX + MARST+ INCOME + EDUS + SMST + DRST + HSUG + LFVI + HSALT + HFAT + SETLR, data = df, family = binomial

	(link = "logit"), id = id, corstr = "independence", scale.fix = TRUE, scale.value = 1)<br><br>gee (UR ~ INDEX + SEX + AGE + MARST+ INCOME + EDUS + SMST + DRST + HSUG + LFVI + HSALT + HFAT + SETLR, data = df, family = binomial (link = "logit"), id = id, corstr = "independence", scale.fix = TRUE, scale.value = 1)<br><br>gee (OB ~ INDEX + SEX + AGE + MARST+ INCOME + EDUS + SMST + DRST + HSUG + LFVI + HSALT + HFAT + SETLR, data = df, family = binomial (link = "logit"), id = id, corstr = "independence", scale.fix = TRUE, scale.value = 1)

Table S15: R code for men.
Outcomes: Categorical variables – AG, PREAG, SD, PRESD, GFR, UR, OB.

Men	
Deprivation index	
Model0	gee (AG ~ INDEX , data = df, family = binomial (link = "logit"), id = id, corstr = "independence", scale.fix = TRUE, scale.value = 1)<br><br>gee (PREAG ~ INDEX , data = df, family = binomial (link = "logit"), id = id, corstr = "independence", scale.fix = TRUE, scale.value = 1)<br><br>gee (PRESD ~ INDEX , data = df, family = binomial (link = "logit"), id = id, corstr = "independence", scale.fix = TRUE, scale.value = 1)<br><br>gee (SD ~ INDEX , data = df, family = binomial (link = "logit"), id = id, corstr = "independence", scale.fix = TRUE, scale.value = 1)<br><br>gee (GFR ~ INDEX , data = df, family = binomial (link = "logit"), id = id, corstr = "independence", scale.fix = TRUE, scale.value = 1)<br><br>gee (UR ~ INDEX , data = df, family = binomial (link = "logit"), id = id, corstr = "independence", scale.fix = TRUE, scale.value = 1)<br><br>gee (OB ~ INDEX , data = df, family = binomial (link = "logit"), id = id, corstr = "independence", scale.fix = TRUE, scale.value = 1)
Model1	gee (AG ~ INDEX + SETLR + SMST + EDUS + MARST + AGE, data = df, family = binomial (link = "logit"), id = id, corstr = "independence", scale.fix = TRUE, scale.value = 1)<br><br>gee (PREAG ~ INDEX + DRST + MARST + AGE, data = df, family = binomial (link = "logit"), id = id, corstr = "independence", scale.fix = TRUE, scale.value = 1)<br><br>gee (PRESD ~ INDEX + EDUS + INCOME + MARST + AGE, data = df, family = binomial (link = "logit"), id = id, corstr = "independence", scale.fix = TRUE, scale.value = 1)

	gee (SD ~ INDEX + LFVI + HSUG + DRST + EDUS + INCOME + MARST + AGE, data = df, family = binomial (link = "logit"), id = id, corstr = "independence", scale.fix = TRUE, scale.value = 1)<br><br>gee (GFR ~ INDEX + SETLR + LFVI + DRST + SMST + EDUS + INCOME, data = df, family = binomial (link = "logit"), id = id, corstr = "independence", scale.fix = TRUE, scale.value = 1)<br><br>gee (UR ~ INDEX + SETLR + DRST + SMST + EDUS + INCOME + MARST + AGE, data = df, family = binomial (link = "logit"), id = id, corstr = "independence", scale.fix = TRUE, scale.value = 1)<br><br>gee (OB ~INDEX + SETLR + LFVI + HSUG + DRST + SMST + EDUS + MARST + AGE, data = df, family = binomial (link = "logit"), id = id, corstr = "independence", scale.fix = TRUE, scale.value = 1)
Model2	gee (AG ~ INDEX + AGE + MARST+ INCOME + EDUS + SMST + DRST + HSUG + LFVI + HSALT + HFAT + SETLR, data = df, family = binomial (link = "logit"), id = id, corstr = "independence", scale.fix = TRUE, scale.value = 1)<br><br>gee (PREAG ~ INDEX + AGE + MARST+ INCOME + EDUS + SMST + DRST + HSUG + LFVI + HSALT + HFAT + SETLR, data = df, family = binomial (link = "logit"), id = id, corstr = "independence", scale.fix = TRUE, scale.value = 1)<br><br>gee (PRESD ~ INDEX + AGE + MARST+ INCOME + EDUS + SMST + DRST + HSUG + LFVI + HSALT + HFAT + SETLR, data = df, family = binomial (link = "logit"), id = id, corstr = "independence", scale.fix = TRUE, scale.value = 1)<br><br>gee (SD ~ INDEX + AGE + MARST+ INCOME + EDUS + SMST + DRST + HSUG + LFVI + HSALT + HFAT + SETLR, data = df, family = binomial (link = "logit"), id = id, corstr = "independence", scale.fix = TRUE, scale.value = 1)<br><br>gee (GFR ~ INDEX + MARST+ INCOME + EDUS + SMST + DRST + HSUG + LFVI + HSALT + HFAT + SETLR, data = df, family = binomial (link = "logit"), id = id, corstr = "independence", scale.fix = TRUE, scale.value = 1)<br><br>gee (UR ~ INDEX + AGE + MARST+ INCOME + EDUS + SMST + DRST + HSUG + LFVI + HSALT + HFAT + SETLR, data = df, family = binomial (link = "logit"), id = id, corstr = "independence", scale.fix = TRUE, scale.value = 1)<br><br>gee (OB ~ INDEX + AGE + MARST+ INCOME + EDUS + SMST + DRST + HSUG + LFVI + HSALT + HFAT + SETLR, data = df, family = binomial (link = "logit"), id = id, corstr = "independence", scale.fix = TRUE, scale.value = 1)

Table S16: R code for women.
Outcomes: Categorical variables – AG, PREAG, PRESD, SD, GFR, UR, OB.

Women	
Deprivation index	
Model0	gee (AG ~ INDEX , data = df, family = binomial (link = "logit"), id = id, corstr = "independence", scale.fix = TRUE, scale.value = 1)<br><br>gee (PREAG ~ INDEX , data = df, family = binomial (link = "logit"), id = id, corstr = "independence", scale.fix = TRUE, scale.value = 1)<br><br>gee (PRESD ~ INDEX , data = df, family = binomial (link = "logit"), id = id, corstr = "independence", scale.fix = TRUE, scale.value = 1)<br><br>gee (SD ~ INDEX , data = df, family = binomial (link = "logit"), id = id, corstr = "independence", scale.fix = TRUE, scale.value = 1)<br><br>gee (GFR ~ INDEX , data = df, family = binomial (link = "logit"), id = id, corstr = "independence", scale.fix = TRUE, scale.value = 1)<br><br>gee (UR ~ INDEX , data = df, family = binomial (link = "logit"), id = id, corstr = "independence", scale.fix = TRUE, scale.value = 1)<br><br>gee (OB ~ INDEX , data = df, family = binomial (link = "logit"), id = id, corstr = "independence", scale.fix = TRUE, scale.value = 1)
Model1	gee (AG ~ INDEX + SETLR + HSALT + HSUG + DRST + SMST + EDUS + INCOME + MARST + AGE, data = df, family = binomial (link = "logit"), id = id, corstr = "independence", scale.fix = TRUE, scale.value = 1)<br><br>gee (PREAG ~ INDEX + SETLR + DRST + SMST + EDUS + INCOME + MARST + AGE, data = df, family = binomial (link = "logit"), id = id, corstr = "independence", scale.fix = TRUE, scale.value = 1)<br><br>gee (PRESD ~INDEX + SETLR + HSUG + DRST + SMST + EDUS + INCOME + MARST + AGE , data = df, family = binomial (link = "logit"), id = id, corstr = "independence", scale.fix = TRUE, scale.value = 1)<br><br>gee (GFR ~ INDEX + SETLR + HSUG + SMST + INCOME, data = df, family = binomial (link = "logit"), id = id, corstr = "independence", scale.fix = TRUE, scale.value = 1)<br><br>gee (UR ~ INDEX + SETLR + HSUG + DRST + SMST + EDUS + INCOME + MARST + AGE, data = df, family = binomial (link = "logit"), id = id, corstr = "independence", scale.fix = TRUE, scale.value = 1)<br><br>gee (OB ~ INDEX + SETLR + DRST + SMST + EDUS + INCOME + MARST + AGE, data = df, family = binomial (link = "logit"), id = id, corstr = "independence", scale.fix = TRUE, scale.value = 1)
Model2	gee (AG ~ INDEX + AGE + MARST+ INCOME + EDUS + SMST + DRST + HSUG + LFVI + HSALT + HFAT + SETLR, data = df, family = binomial (link = "logit"), id = id, corstr = "independence", scale.fix = TRUE, scale.value = 1)

	gee (PREAG ~ INDEX + AGE + MARST+ INCOME + EDUS + SMST + DRST + HSUG + LFVI + HSALT + HFAT + SETLR, data = df, family = binomial (link = "logit"), id = id, corstr = "independence", scale.fix = TRUE, scale.value = 1)<br><br>gee (SD ~ INDEX + LFVI + HSUG + DRST + SMST + INCOME + MARST + AGE, data = df, family = binomial (link = "logit"), id = id, corstr = "independence", scale.fix = TRUE, scale.value = 1)<br><br>gee (PRESD ~ INDEX + AGE + MARST+ INCOME + EDUS + SMST + DRST + HSUG + LFVI + HSALT + HFAT + SETLR, data = df, family = binomial (link = "logit"), id = id, corstr = "independence", scale.fix = TRUE, scale.value = 1)<br><br>gee (SD ~ INDEX + AGE + MARST+ INCOME + EDUS + SMST + DRST + HSUG + LFVI + HSALT + HFAT + SETLR, data = df, family = binomial (link = "logit"), id = id, corstr = "independence", scale.fix = TRUE, scale.value = 1)<br><br>gee (GFR ~ INDEX + MARST+ INCOME + EDUS + SMST + DRST + HSUG + LFVI + HSALT + HFAT + SETLR, data = df, family = binomial (link = "logit"), id = id, corstr = "independence", scale.fix = TRUE, scale.value = 1)<br><br>gee (UR ~ INDEX + AGE + MARST+ INCOME + EDUS + SMST + DRST + HSUG + LFVI + HSALT + HFAT + SETLR, data = df, family = binomial (link = "logit"), id = id, corstr = "independence", scale.fix = TRUE, scale.value = 1)<br><br>gee (OB ~ INDEX + AGE + MARST+ INCOME + EDUS + SMST + DRST + HSUG + LFVI + HSALT + HFAT + SETLR, data = df, family = binomial (link = "logit"), id = id, corstr = "independence", scale.fix = TRUE, scale.value = 1)

Table S17: R code for total population, men and women.
Outcomes: Continuous variables – SBP, DBP, UACIDE, CRE, GLU, IBM.

Total population
gee(SBP ~ INDEX + AGE + SEX + AHTRT + INCOME + EDUS + DRST + SMST + HSUG + LFVI + HSALT + HFAT + SETLR + MARST, data = df, family = gaussian(link = "identity"),id = id, corstr = "independence")<br><br>gee(DBP ~ INDEX + AGE + SEX + AHTRT + INCOME + EDUS + DRST + SMST + HSUG + LFVI + HSALT + HFAT + SETLR + MARST, data = df, family = gaussian(link = "identity"),id = id, corstr = "independence")<br><br>gee(UACIDE~ INDEX + AGE + SEX + INCOME + EDUS + DRST + SMST + HSUG + LFVI + HSALT + HFAT + SETLR + MARST, data = df, family = gaussian(link = "identity"),id = id, corstr = "independence")

gee(CRE ~ INDEX + AGE + SEX + INCOME + EDUS + DRST + SMST + HSUG + LFVI + HSALT + HFAT + SETLR + MARST, data = df, family = gaussian(link = "identity"),id = id, corstr = "independence")<br><br>gee(GLU~ INDEX + AGE + SEX + DMDR2W +INCOME + EDUS + DRST + SMST + HSUG + LFVI + HSALT + HFAT + SETLR + MARST, data = df, family = gaussian(link = "identity"),id = id, corstr = "independence")<br><br>gee(IBM~ INDEX + AGE + SEX + INCOME + EDUS + DRST + SMST + HSUG + LFVI + HSALT + HFAT + SETLR + MARST, data = df, family = gaussian(link = "identity"),id = id, corstr = "independence")
Men/women
gee(SBP ~ INDEX + AGE + AHTRT + INCOME + EDUS + DRST + SMST + HSUG + LFVI + HSALT + HFAT + SETLR + MARST, data = df, family = gaussian(link = "identity"),id = id, corstr = "independence")<br><br>gee(DBP ~ INDEX + AGE + AHTRT + INCOME + EDUS + DRST + SMST + HSUG + LFVI + HSALT + HFAT + SETLR + MARST, data = df, family = gaussian(link = "identity"),id = id, corstr = "independence")<br><br>gee(UACIDE~ INDEX + AGE + INCOME + EDUS + DRST + SMST + HSUG + LFVI + HSALT + HFAT + SETLR + MARST, data = df, family = gaussian(link = "identity"),id = id, corstr = "independence")<br><br>gee(CRE ~ INDEX + AGE + INCOME + EDUS + DRST + SMST + HSUG + LFVI + HSALT + HFAT + SETLR + MARST, data = df, family = gaussian(link = "identity"),id = id, corstr = "independence")<br><br>gee(GLU~ INDEX + AGE + DMDR2W +INCOME + EDUS + DRST + SMST + HSUG + LFVI + HSALT + HFAT + SETLR + MARST, data = df, family = gaussian(link = "identity"),id = id, corstr = "independence")<br><br>gee(IBM~ INDEX + AGE + INCOME + EDUS + DRST + SMST + HSUG + LFVI + HSALT + HFAT + SETLR + MARST, data = df, family = gaussian(link = "identity"),id = id, corstr = "independence")
